# Overcoming high-temperature performance limitations: Multidimensional design and innovation of thin-film thermocouples

**DOI:** 10.1016/j.isci.2026.116209

**Published:** 2026-06-08

**Authors:** Zhongkai Zhang, Tianci Zhang, Zhaojun Liu, Meng Wang, Guoliang Zhou, Xiang Liu, Chen Wu, Xudong Fang, Duanzhi Duan, Bian Tian

**Affiliations:** 1 State Key Laboratory for Manufacturing Systems Engineering, International Joint Laboratory for Micro/Nano Manufacturing and Measurement Technologies, School of Mechanical Engineering, Xi’an Jiaotong University, Xi’an 710049, China

**Keywords:** Classification Description, thermodynamics, applied sciences, mechanical engineering

## Abstract

Thin-film thermocouples (TFTCs) are known for their thinness, low thermal mass, fast response time, and low interference with component structure and flow field. High-temperature sensors have become an essential technological area for high-temperature tests of advanced equipment such as aeroengines and hypersonic vehicles. This paper presents a systematic review of research advancements in high-temperature thin-film thermocouples (HT-TFTCs) across five core dimensions: material systems, structural design, fabrication processes, failure mechanisms and protection, and self-healing technologies. In addition, this paper outlines future development trends. In order to enhance the development of HT-TFTCs with long service life and high reliability, as well as intelligent applications, it is necessary to strengthen multi-dimensional collaborative design and integrated innovation, further promote self-healing technology with intelligent regeneration, and overcome performance limitations.

## Introduction

Technologies such as aerospace engineering and energy propulsion systems have gained growing global attention with 21st century developments. The temperature monitoring for demanding high-temperature conditions has emerged as a key factor impacting performance, safety, and design.^1^^,^^2^ Many temperature measurement devices, especially those which utilize contact methods like sheathed wire thermocouples, have been improved and are still regularly employed in industry. However, these devices have been unable to adapt to high-performance aerospace propulsion systems, hypersonic vehicles, as well as large-scale gas turbines. There are several limitations of these sensors. The mass and size of the armored thermocouple significantly change the shape, resistance, and temperature of the object being measured, resulting in distorted measurement data.^3^^,^^4^ Furthermore, stresses are introduced at the installation site by the armored thermocouple.^3^ In addition, thermal inertia leads to delays in the signal, making it impossible to track dynamic processes accurately.^5^^,^^6^^,^^7^

To overcome the drawbacks, High-temperature thermoelectric sensors based on thin-film technology have been developed, which is a substantial advancement in thermodynamic measurement. Through micro- and nanofabrication procedures, the thermoelectric material films are directly deposited at the micrometer or nanometer scale onto the surface of the target object. This method greatly decreases both the thickness and mass of the sensor and eliminates the need for any mechanical drilling, thereby minimizing interaction with the mechanical properties, fluid characteristics, and heat properties of the measured part. These sensors can respond within milliseconds to microseconds because their thermal inertia is very low. Thus, they can measure rapid changes in temperature perfectly. As a result, this effect significantly improves the timeliness and credibility of measurement data.

**Figure 1:**
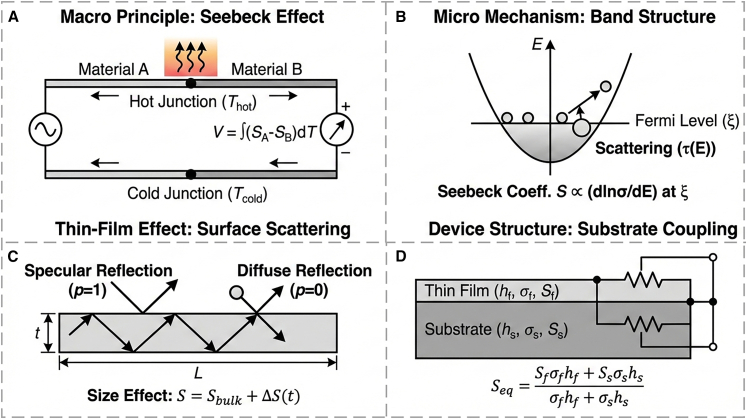
Schematic diagrams illustrating the principles of HT-TFTCs (A) The Seebeck effect. (B) Fermi level and electronic density of states. (C) Size effects and scattering models. (D) Substrate coupling and the bilayer model.

HT-TFTCs function based on the Seebeck effect (Figure 1A). A thermoelectric potential is generated within a closed circuit formed by two dissimilar conductors or semiconductors when a temperature gradient exists between the two junctions. The magnitude of this potential is contingent upon the material properties and demonstrates a linear relationship with the temperature difference, facilitating temperature inversion through potential detection. The relationship between thermoelectric potential and temperature difference is represented by Equation 1:^8^

(Equation 1) V=SΔT

where *V* is the thermoelectric potential, *S* is the Seebeck coefficient, and *T* is the temperature difference. The Seebeck coefficient is an intrinsic thermoelectric property of the material, which strongly depends on the electron density of states near the Fermi level and the scattering mechanisms. Within the free-electron model, the thermoelectric power for bulk material made of pure metal is given by Equation 2:^9^

(Equation 2) $$S_{B}=-\frac{\pi^{2}}{3e}\frac{kT}{\xi }\left(U+V\right)$$

where $U={\left(\frac{\partial \ln\;l}{\partial \ln\;E}\right)}_{E=\xi }$ represents the degree of dependence of the average free path *l* on energy *E*, $V={\left(\frac{\partial \ln\;A}{\partial \ln\;E}\right)}_{E=\xi }$ represents the degree of dependence of the Fermi surface area *A* on energy *E*, *ξ* denotes the Fermi level, *T* is the temperature, *e* is the absolute value of the electron charge, and *k* is the Boltzmann constant.

Under the free-electron approximation, *V* = 1; in contrast, according to Bloch quantum theory, *U* = 2 (i.e., *l* ∝ *E*^2^). However, actual measurements show that precious metals such as Cu exhibit a positive Seebeck coefficient, contradicting the negative value predicted by free-electron theory. This effect indicates the need to consider band structure and inelastic scattering. As illustrated in Figure 1B, the Fermi surface of Cu contacts the Brillouin zone boundaries, resulting in a decrease in the Fermi surface area with increasing energy.

In thin-film architectures, thermoelectric performance is significantly governed by size effects and substrate interfacial effects. Figure 1C depicts the electron trajectories within the film, demonstrating that electrons undergo scattering not only from the crystal lattice but also at the upper and lower surfaces of the film. When the film thickness *t* is comparable to the carrier’s mean free path *l*, surface scattering alters transport processes. According to the size effect theory derived from the free-electron model, the thermoelectric power *S*_*P*_ of a film can be expressed as Equation 3:

(Equation 3) $$S_{P}=S_{B}\left[1-\frac{3}{8}\frac{l}{t}\frac{\left(1-p\right)U}{1+U}\right],\left(t>l\right)$$

where *p* is the surface specular scattering coefficient (*p* ≈ 0 for polycrystalline films). The variation of thermoelectric power *ΔS*_*F*_ is inversely proportional to thickness. Equation 4 demonstrates their relationship:

(Equation 4) $$\Delta S_{F}=S_{B}-S_{P}=S_{B}\frac{3}{8}\frac{l\left(1-p\right)}{t}\frac{U}{1+U}=-\frac{\pi^{2}}{8e}\frac{kT}{\xi }\frac{l\left(1-p\right)U}{t}$$

Furthermore, since thin films are invariably deposited on substrates for practical applications, the substrate modulates the overall thermoelectric response through electrical and thermal coupling within the multilayer structure. As shown in Figure 1D, the HT-TFTCs can be modeled as a voltage source, while a conductive substrate (e.g., a silicon wafer) acts as a parallel resistor. Consequently, the equivalent Seebeck coefficient can be calculated using the bilayer model:^10^

(Equation 5) $$S_{eq}=\frac{h_{\text{sub}}\sigma_{\text{sub}}S_{\text{sub}}+h_{\text{thermo}.}\sigma_{\text{thermo}.}S_{\text{thermo}.}}{h_{\text{sub}}\sigma_{\text{sub}}+h_{\text{thermo}.}\sigma_{\text{thermo}.}}$$

In the context of HT-TFTCs, the relationship between film thickness (*h*) and electrical conductivity (*σ*) is critical. Equation 5 demonstrates that as film thickness decreases, the substrate contribution to the measured Seebeck coefficient increases, leading to a deviation from the value observed in bulk materials, especially at lower temperatures. Therefore, the performance of HT-TFTCs is influenced not only by the intrinsic Seebeck coefficient of the material but also by thickness-related size effects and substrate-interface interactions. Consequently, effective design and application necessitate an optimization of film thickness and structure to achieve a balance between sensitivity, response time, and stability.

**Figure 2:**
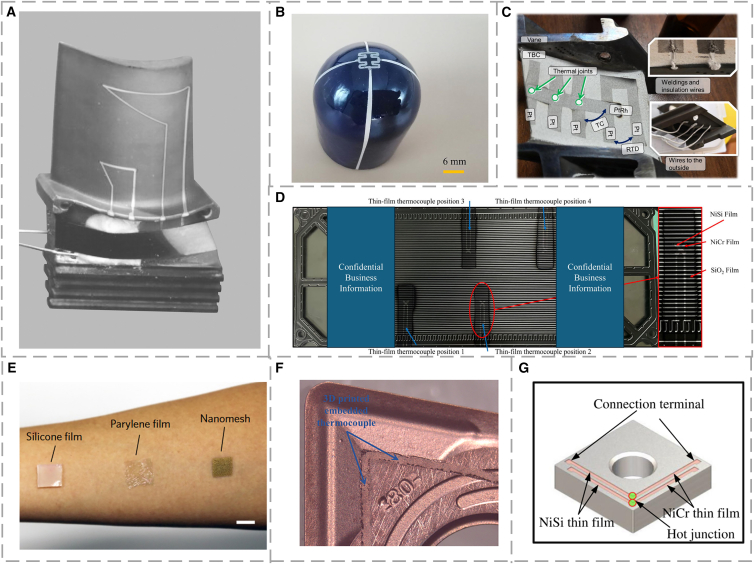
Applications of HT-TFTCs in diverse fields (A) Integration on aeroengine turbine blade. Reprinted from Matus et al.,^11^ with the permission of ASCE Publications. (B) Spherical thin-film temperature sensor. Reprinted from Xu et al.,^12^ with the permission of Elsevier Publishing. (C) HT-TFTC array. Reprinted from Duan et al.,^13^ with the permission of AIAA Publications. (D) HT-TFTCs for fuel cell temperature measurement. Reprinted from Guo et al.,^14^ with the permission of MDPI Publishing. (E) Flexible thin-film sensors on human skin. Reprinted from Miyamoto et al.,^15^ with the permission of Nature Publishing. (F) HT-TFTCs on cutting tool surface. Reprinted from Guimarães et al.,^16^ with the permission of Elsevier Publishing. (G) Schematic of HT-TFTC structure on cutting tool surface. Reprinted from Chen et al.,^17^ with the permission of Elsevier Publishing.

The technical advantages of these sensors have made them widely useful in various multidisciplinary fields. In 1994, NASA’s Lewis Research Center achieved the successful integration of thin-film thermoelectric sensing elements on the surfaces of aerospace engine blades (Figure 2A).^11^^,^^18^ Recently, a group led by Sun Daoheng in China’s Xiamen University used a conformal insulating coating process for metal substrates and achieved functional films with a surface roughness of 0.15 μm, an insulation strength of 3 MΩ, and an adhesion strength of 55.3 N. Films withstanding extreme heat fluxes of over 1.2 MW m^−2^ are possible (Figure 2B).^12^ The Duan Li research group at Shanghai Jiao Tong University used Micro-Electro-Mechanical Systems (MEMS) technology to fabricate platinum/platinum-rhodium thin-film sensor arrays on turbine guide vane surfaces. The combined test for these arrays under 40 *g* vibration and 1,200°C high-pressure gas was successfully passed (Figure 2C).^13^ Their innovative soft photolithography technique further facilitated the patterned manufacturing of curved blade sensor arrays, providing a novel solution for multi-sensor integration in smart aeroengines.^19^ In industrial applications, Guo et al. from Tongji University utilized HT-TFTCs for *in situ* contact measurements of reaction temperatures in proton exchange membrane fuel cells (Figure 2D).^14^ The Grassini team at Politecnico di Torino developed thin-film thermoelectric arrays that were directly deposited onto freeze-dried pharmaceutical container walls for temperature monitoring during cryogenic processes.^20^ In biomedical applications, the flexible properties and microstructure of HT-TFTCs enable them to adhere closely to human skin, continuously acquiring surface temperature data and thermal field distribution information (Figure 2E).^21^ The breathable, ultra-thin elastic sensor developed by Miyamoto demonstrates biocompatibility, allowing for long-term attachment to skin surfaces without causing irritation.^15^ During manufacturing, significant thermal energy is released at the tool-workpiece contact interface due to friction. This thermal effect accelerates material wear and has a substantial impact on tool life (Figure 2F).^16^ Consequently, the dynamic monitoring of tool surface temperatures using HT-TFTCs establishes a basis for optimizing machining parameters, facilitating cost control, extending tool service life, and enhancing machining efficiency (Figure 2G).^17^^,^^22^

The development of HT-TFTCs represents a classic example of interdisciplinary collaboration. The materials science and solid-state physics community strives to screen functional candidates and understand thermoelectric transport mechanisms to maximize sensitivity. At the same time, heat transfer and solid mechanics can analyze transient thermal response and stress evolution to ensure integrity in extreme conditions. The theory and application link micro/nano-manufacturing, which resolves the deposition and conformal patterning challenges on complex curvilinear topologies.

Most literature is on general overview and specific research directions of HT-TFTCs. This review presents a multi-dimensional systems analysis to examine five dimensions simultaneously which are material composition, configuration optimization, processing techniques, damage mechanisms and protection measures, and self-repairing methods. The design dimensions are somewhat independent but can be interdependent and collectively operate on the complete design process starting from device design and manufacturing to the performance maintenance and functional recovery. This paper integrates the research trends of HT-TFTCs in many disciplines and forecasts future trends for cross-disciplinary collaborative developments.

## Materials of HT-TFTCs

As the thrust-to-weight ratio of aero-engines increases, the combustion chamber’s exhaust temperatures have raised above 2,000°C leading to thermal shock and erosion due to high-velocity airflow.^23^^,^^24^^,^^25^ Consequently, there is a continual need for progressive development of HT-TFTC materials as their choice affects the performance of the device. Since the 1970s, NASA has been developing various noble metal-based thermocouple wires. These wire types and their usage and benefits are T type, K type, S type, and R type. Additionally, they are chosen through experimentation due to their high conductivity and thermal stability.^26^^,^^27^^,^^28^^,^^29^^,^^30^^,^^31^^,^^32^

**Figure 3:**
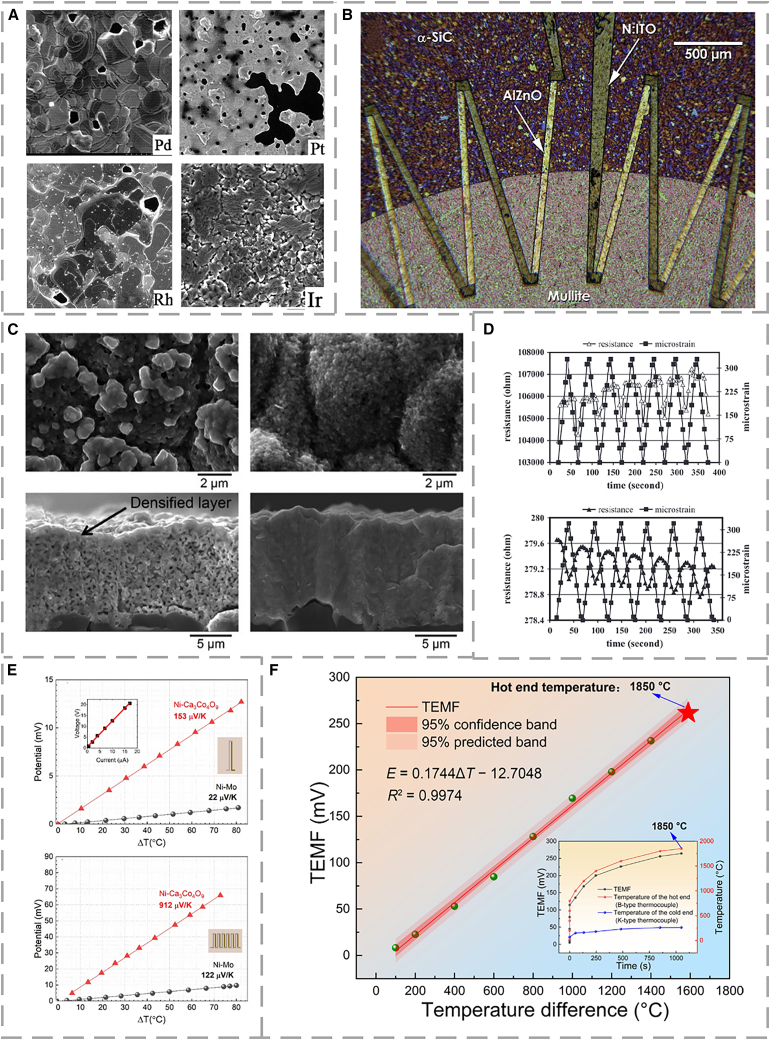
Material advancements in HT-TFTCs (A) SEM images of Pt, Pd, Rh, and Ir thin films. Reprinted from Kreider et al.,^39^ with the permission of Elsevier Publishing. (B) In_2_O_3_-ZnO composite ceramic thin films. Reprinted from Wrbanek et al.,^40^ with the permission of Elsevier Publishing. (C) SEM micrographs of InON and In_2_O_3_ films and their fracture cross-sections. Reprinted from Gregory et al.,^41^ with the permission of Wiley-Blackwell Publishing. (D) Piezoresistive response of ITO films before and after doping. Reprinted from Gregory et al.,^42^ with the permission of Elsevier Publishing. (E) The outputs of Ni–Mo and Ni-Ca_3_Co_4_O_9_ thermocouples with temperature gradient (ΔT). Reprinted from Xin et al.,^43^ with the permission of Elsevier Publishing. (F) Thermoelectric characteristic curve of the ultra-high-temperature test. Reprinted from Wang et al.,^44^ with the permission of Journal of Advanced Ceramics.

At the same time, Kreider’s NIST team assessed high-temperature stability of non-standard Pd, Pt, Rh, and Ir films.^33^^,^^34^^,^^35^^,^^36^^,^^37^^,^^38^ As shown in Figure 3A, Pd films annealed for 2 h at 900°C developed significant voids and grain growth, resulting in a drift of 9% at 880°C that limits their usability to about 850°C. The Ti adhesion layer of the Pt films underwent migration and oxidation between 950°C and 1,000°C, causing adhesion loss and delamination. On the contrary, the Rh films showed good stability when annealed 1,000°C with thermoelectric hysteresis less than 0.6%. Ir films showed the best performance with hysteresis less than 0.1% and adhesion strength more than 50 MPa. As a result, the Rh/Ir setup is the most appropriate for use above 900°C owing to their excellent resistance to coalescence and diffusion and good thermoelectric stability.^39^

With advances in ceramic technology, ceramics have emerged as one of the most extensively studied materials due to their exceptional high-temperature oxidation resistance, chemical inertness, and thermoelectric properties, which surpass those of precious metals.^45^ In 2012, Wrbanek et al. from NASA manufactured a finely lined aluminum zinc oxide-nitrogen:doped indium tin oxide (AlZnO-N:ITO) thermopile via sputter deposition with a partial mullite overcoat on disk substrates. As can be seen in Figure 3B, this structure displayed stable operation at 800°C for 150 min.^40^ Liu et al. fabricated three oxide HT-TFTCs by radio frequency magnetron sputtering (RF magnetron sputtering).^46^ The authors showed experimentally that In_2_O_3_/Pt produced a Seebeck coefficient of 203.9 μV °C^−1^ at 900°C temperature difference or 3 times superior performance compared to metallic thermocouples. Similarly, the ITO/In_2_O_3_ system had a very stable voltage output of 165.7 mV at 1,200°C (R^2^ > 0.999).

Advancements in MEMS fabrication and nanodoping have shifted the operating limit of ceramic HT-TFTCs to 1,450°C–1,500°C with ultrafast response in less than 1.5 ms, outperforming metal ones by a large margin.^47^^,^^48^^,^^49^^,^^50^^,^^51^^,^^52^ Still, a significant challenge lies with the volatility of elements such as Sn and stability of oxygen vacancies at high temperature. Specifically, reduction reactions that form volatile species are responsible for oxide materials. The resulting Sn loss (the main carrier source in ITO) reduces carrier concentration and influences electrical properties. As a result of this degradation mechanism, significant drift in the thermoelectric output signal occurs.^53^

Gregory et al. utilized ITO targets to sputter-deposit In_2_O_3_ films in an Ar/N_2_-mixed atmosphere, thereby addressing the aforementioned challenges.^41^ As shown in Figure 3C, the nitrogen-treated InON film has a dense surface oxynitride layer and an internally connected submicrometer pore structure. On the other hand, the In_2_O_3_ film, sputtered in pure Ar, has completely dense microstructure. This thick surface layer effectively prevents the diffusion of oxygen from the atmosphere into the bulk material, while the internal porous structure prevents sintering and excessive grain growth due to high temperature. As a result, the ongoing stabilization of oxygen vacancies leads to the preservation of a zero Seebeck coefficient with increased temperature. It is remarkable that the InON scheme maintains its structure at 1,500°C, and Sn volatilization can be prevented.^45^^,^^54^

In their study on aluminum doping, Gregory et al. illustrated significant findings, as shown in Figure 3D.^42^ The undoped ITO thin-film strain sensor approached failure at a temperature of 1,225°C, which was characterized by a sharp surge in resistance (above 100 kΩ), severe degradation of the waveform, and notable baseline drift. Electrical instability occurs due to excessive compensation of oxygen vacancies and reduced decomposition at high temperatures. In comparison, an aluminum-doped ITO film of the same thickness (optimized for the Al/In ratio at low temperature with post-annealing in a partial oxygen atmosphere) exhibited a strong piezoresistive response at an even higher temperature of 1,280°C. This device exhibited a gauge factor equal to 4.50. It has a drift rate of −0.0098% h^−1^. Moreover, it has a stable response. As this comparison shows, suitable aluminum doping markedly improves the electrical and thermoelectric stability of ITO in ultra-high-temperature conditions due to the formation of stable interfacial reaction products and suppression of oxygen diffusion. A comprehensive analysis reveals that the combined actions of doping and microstructure regulation can effectively alleviate the dual challenges of oxygen diffusion and component volatilization, thus significantly enhancing their high-temperature performance. Nonetheless, the current methods commonly result in side effects such as decreased electrical conductivity or thermoelectric conversion efficiency.^55^ Hence, the optimization of this balance is a key focus in the future. Furthermore, ceramic films are quite brittle in nature, which makes reliability at the metal/ceramic interface during thermal cycling a limiting factor for long-term stable device operation.

Development of alternative multi-component material systems substituting ITO is emerging to address the demanding requirements of different application scenarios.^56^^,^^57^^,^^58^^,^^59^^,^^60^ In 2023, Xin et al. reportedly developed Ni-Ca_3_Co_4_O_9_ composite HT-TFTC materials.^43^ As shown in Figure 3E, the Seebeck effect voltage measured in this material system is significantly higher than that measured for conventional Ni-Mo thermocouples. The voltage measured for a single pair thermocouple is 153 μV K^−1^ and for a 6-pair series structure is 912 μV K^−1^, resulting in the peak output of 70 mV at a hot-end operating temperature of 105°C. Wang et al. implemented a synergistic approach by combining yttria-stabilized zirconia (YSZ)-modified In_2_O_3_ with an Al_2_O_3_ protective layer and neural network error correction,^44^ successfully raising the maximum operating temperature of HT-TFTCs to 1,850°C, as shown in Figure 3F. In addition, the novel polymer-derived ceramic (PDC) composite systems were developed to allow flexibility in structural design and to improve thermal stability. The ceramic maximum temperature sensors were developed through molecular control of precursor composition and micro-composite techniques.

At present, the manufacturing processes of widely used PDC are very complicated and lack process reproducibility. The ceramic and free carbon phases have an energy band mismatch that inhibits effective enhancement of the Seebeck coefficient. When the temperature rises, nanoscale structural reconstruction will occur as carbon clusters rearrange and crystallization occurs, causing serious changes in electrical transport properties.^61^ Under ultra-high-temperature scenarios, in fact, thermodynamic decomposition of carbon may happen in the material.^62^ Additionally, strong coupling of performance parameters with precursor processing requires strict control, which is a significant bottleneck to industrial application.

Traditional precious metals have repeatedly demonstrated high-temperature adaptability through experimentation. On the other hand, newer ceramic materials have sidestepped all of this. They have taken giant strides forward in recent times in all important parameters, such as high-temperature endurance, thermoelectric conversion efficiency, and response sensitivity. When the Seebeck coefficient reaches a certain level, there is increased defect sensitivity and lower stability of HT-TFTCs. The coming technological advances in this area will also depend on advances in materials. To design the next set of successful electronic devices, multi-scale collaborative design strategies will be required. These strategies will involve atomic-level doping control, micro- and nano-structure construction, and interface optimization techniques. This paradigm shift must move away from traditional material screening to one based on a systematically integrated design, ensuring stable, precise, and long-lasting performance of HT-TFTCs under stress.

## Structural design and optimization of HT-TFTCs

The application of HT-TFTCs to aircraft engine blades can carry a range of challenges such as electrical insulation between the substrate and thermoelectric functional layers,^23^^,^^63^^,^^64^ strong adhesion between the functional layers,^65^ and flexibility of measurements.^66^ Their performance, longevity, and environmental tolerance primarily depend on their architecture. Additionally, applying these devices in harsher surroundings such as aircraft engines and power applications is driving massive demand for structural developments and technological improvements. The construction of these thermocouples does not simply occur by stacking the sensor materials and electrodes. It consists of a precise set of multiple thin-film layers, including a substrate, bonding media, an insulating film, a thermoelectric conversion layer, and a protection coating.

**Figure 4:**
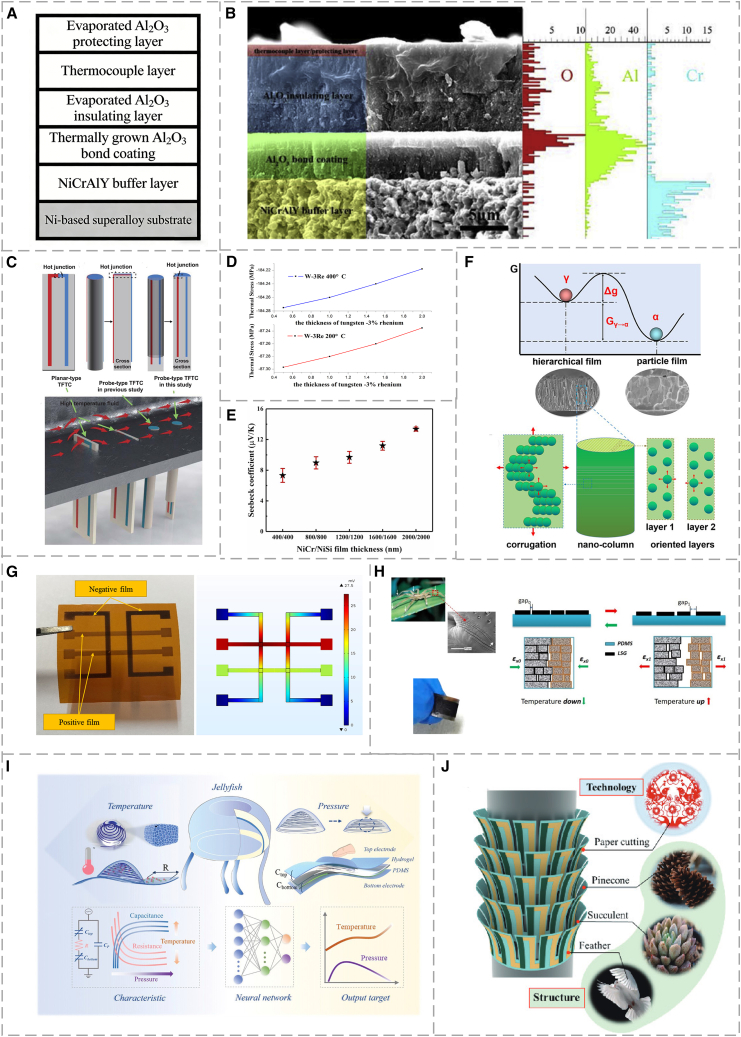
Structural design and optimization of HT-TFTCs (A) Schematic of the “sandwich” architecture. Reprinted from Zhao et al.,^67^ with the permission of Elsevier Publishing. (B) Cross-sectional SEM image and Energy-Dispersive X‑ray spectroscopy (EDX) analysis of an HT-TFTC. Reprinted from Zhao et al.,^67^ with the permission of Elsevier Publishing. (C) Structural configurations and practical applications of cylindrical HT-TFTCs. Reprinted from Fan et al.,^68^ with the permission of IOP Publishing. (D) Variation of thermal stress as a function of temperature and W-3Re film thickness. Reprinted from Zhang et al.,^69^ with the permission of MDPI Publishing. (E) Seebeck coefficients of NiCr/NiSi thermoelectric electrodes with varying thicknesses. Reprinted from Liu et al.,^70^ with the permission of Elsevier Publishing. (F) Schematic diagram of hierarchical Ir nanostructure-based thin films. Reprinted from Luo et al.,^71^ with the permission of ACS Publications. (G) Four-point conjugate structure. Reprinted from Tian et al.,^72^ with the permission of AIP Publishing. (H) Schematic diagrams illustrating the sensing mechanism of crack-based graphene temperature sensors. Reprinted from Tian et al.,^73^ with the permission of RSC Advances. (I) Jellyfish-inspired sensor device schematic. Reprinted from Ren et al.,^74^ with the permission of Wiley. (J) Schematic illustration showing the configuration of flexible thermoelectric generators (F-TEGs). Reprinted from Liu et al.,^75^ with the permission of Springer Nature.

Developments of HT-TFTC two-dimensional prototype can be traced back to the research of German researcher Hackenann during World War II. Of all the different constructions, the “sandwich” structure formed the bulk of the standard, most notably Jiang et al.’s multilayer composite. As shown in Figure 4A, all functional layers provide necessary physical properties in this complex design.^67^^,^^76^^,^^77^

The NiCrAlY bond layer is selected because it has matching coefficients of thermal expansion (CTEs) to Ni-based superalloy substrates, providing good bonding with turbine blades. Significantly, the thermally grown oxide Al_2_O_3_ layer, which is created *in situ* upon oxidation, forms a metallurgical bond with the sub-layer and increases the interfacial shear strength to more than 85 MPa. Figure 4B depicts the microstructure of each layer, all of which display a dense structure. The gradual transition from metal to oxide ceramic layer prevents thermal stress between different layers. In 2024, Fan et al. developed a cylindrical embedded architecture, as shown in Figure 4C. The sensitive film is placed in the axial plane of the structure, and the high-temperature medium only contacts the terminal end.^68^ Aerothermal testing showed that the cylinder withstood for 300 s and that the planar HT-TFTCs delaminated in 30 s due to airflow scouring. This design achieved a thermal response time of 1.8 ms, which is eight times faster than the 14.6 ms of the planar ones, and reduced the maximum thermal stress from 480 to 210 MPa, substantially decreasing failure caused by aerodynamic erosion.

The sandwich structure remains a benchmark engineering solution because it effectively balances reliability, response speed, and process compatibility. The encapsulation of the material guarantees outstanding adhesion (greater than 80 MPa) and oxidation resistance (more than 100 h at 1,600°C), along with a millisecond transient response time.^4^^,^^18^^,^^78^^,^^79^^,^^80^ It is considered superior to interdigital or suspended architectures. It is a leading solution in aerospace for NASA, General Electric Company (GE), and others.^66^^,^^79^^,^^80^^,^^81^^,^^82^^,^^83^^,^^84^^,^^85^^,^^86^^,^^87^^,^^88^^,^^89^

After integrating the sensor with the substrate at the macroscale, it is important to analyze how structural dimensional parameters affect device performance. Research shows that thermocouple film thickness significantly affects thermoelectric properties and mechanical stability.^90^^,^^91^ In particular, Zhang et al. found that the thermal stress of W-Re thin films is thickness dependent. Figure 4D shows that thermal stress decreases with increasing film thickness due to the relaxation of stress caused by bending strains in thicker films.^69^ Liu’s research group at the University of Electronic Science and Technology of China obtained NiCr/NiSi HT-TFTCs of different thicknesses to assess the relationship between film thickness and thermoelectric properties.^70^ Moreover, they explained the physical mechanism of the Seebeck coefficient evolution with thickness, as illustrated in Figure 4E. The Seebeck coefficient varies mainly because of the rise in free electron surface density with thickness. Informed by such insight, one may utilize the “thick negative electrode and thin positive electrode” film configuration to optimize the Seebeck coefficient.

To improve the properties of any material, it is essential to make structural changes at a microscopic level. The Luo research team at Beijing University has developed HT-TFTCs based on Ir nanoscale films with hierarchical columnar features. This structure suppresses atomic migration and grain growth at high temperatures while guiding cracks to propagate along tortuous paths, thereby enhancing the film’s hardness, toughness, and high-temperature stability at 1,000°C.^71^^,^^92^^,^^93^

Structural design optimization has proven effective in addressing the inherent error limitations of traditional sensors. He et al. developed double and multi-junction systems that utilize series signals and algorithmic decoupling to achieve cold-junction-free measurement.^94^ Similarly, as shown in Figure 4G, the four-point conjugate HT-TFTC structure introduced by Tian et al. leverages localized multi-node coordination and cross-regulation effects to increase sensitivity from 137.3 to 190.6 μV °C^−1^.^72^ Furthermore, integrating high-density MEMS redundant arrays with fault rejection algorithms significantly enhances fault tolerance. This strategy ensures that the system maintains high precision and robustness even in the event of single-point failures.^95^^,^^96^^,^^97^

Biomimetic innovations designs draw inspiration from biological microstructures, introducing hierarchical interfaces and adaptive mechanisms to address challenges related to stress diffusion and multi-field coupling. In Figure 4H, Li et al. pre-fabricated regular micro-crack arrays (widths of 3–8 μm) on graphene/polyimide substrates. Thermal stress modulates the opening and closing of cracks under high temperatures, resulting in a resistance change rate of 9.93 × 10^−3^ °C^−1^—representing a 390-fold enhancement in sensitivity compared to crack-free structures. The device demonstrated excellent fatigue resistance, with no crack propagation observed after 500 h of continuous operation at 1,200°C.^73^ In Figure 4I, researchers at the Harbin Institute of Technology integrated ion-gel tentacles with columnar thermoelectric arms to achieve simultaneous decoupling of pressure and temperature.^74^ This structure achieved a temperature sensitivity of 4,605 kPa^−1^, with cross-sensitivity below 0.3%, and maintained flexible adhesion in gas flows at 1,300°C while mimicking the gradient gel-skeleton design of jellyfish. Similarly, Liu et al. emulated the suspended three-dimensional morphology of pinecones and feathers to penetrate the laminar boundary layer of high-temperature sources, as illustrated in Figure 4J, achieving a power output of 66.5 mW m^−2^ under a temperature differential of 1,000°C.^75^ Hao et al. drew inspiration from the nacreous layer of seashells to develop an MXene-based thermosensitive elastomer sensor (TES) utilizing a biomimetic lamination strategy that integrates in-plane stress dissipation with a hierarchical architecture. This innovation resulted in a thermal sensitivity of −1.32% °C^−1^, a resolution of approximately 0.3°C, and folding fatigue resistance exceeding 20,000 cycles.^98^ The intrinsic value of these innovative methodologies lies in transcending mere morphological mimicry to extract superior physical principles, which presents vast opportunities for the future development of high-temperature thin-film sensors.

Research on structural innovation and performance enhancement of HT-TFTCs has reported significant advancement. The use of sandwich-type composite layered architectures at early stages has led to improvement of adhesion to substrate and ensured electrical insulation properties. This has enhanced detection accuracy significantly by omitting cold-junction compensation in further novel configurations, leading to biomimetic structural designs for various application scenarios. These technological developments have constantly expanding the detection range and performance parameters of the sensor.

However, the widely adopted multi-material multilayer structures still suffer from complex fabrication processes, numerous interlayer interfaces, and susceptibility to measurement inaccuracies. To address these issues, future research should focus on structural simplification strategies. One promising approach is the adoption of single-material gradient structures, in which the Seebeck effect is generated by thickness or width variations within the same thermoelectric material (e.g., single-metal thermocouples based on Ni or Bi films utilizing size-dependent surface scattering). Another effective pathway is the development of functionally graded materials (FGMs), enabling continuous gradients of thermoelectric and mechanical properties within a single or few-layer film, thereby significantly reducing the number of material types and interfaces. In addition, biomimetic multilevel interface designs can be employed to construct quasi-single-layer multifunctional structures that simultaneously provide sensing, stress dissipation, and adaptive capabilities. These simplification strategies are expected to effectively reduce fabrication complexity, improve long-term reliability and measurement accuracy, and promote the evolution of HT-TFTCs from traditional multilayer stacking toward thinner, more integrated architectures.

## Fabrication processes for HT-TFTCs

Thin-film sensors with high adhesion and uniform thickness are required to be integrated on complex curves for their dynamic temperature monitoring on heavy-duty gas turbine rotor blades to capture instantaneous temperature distribution during rotation. In this context, temperature sensors must withstand operational temperatures of 1,300°C and above while being subjected to centrifugal stresses at thousands of revolutions per minute and exposed to high-temperature combustion gases, which are corrosive in nature.^99^ Deposition of high-quality thin films on curved substrates in a uniform manner is an important process step for sensor development such as HT-TFTCs.^100^ These requirements put extra demands on thin-film fabrication processes.

**Figure 5:**
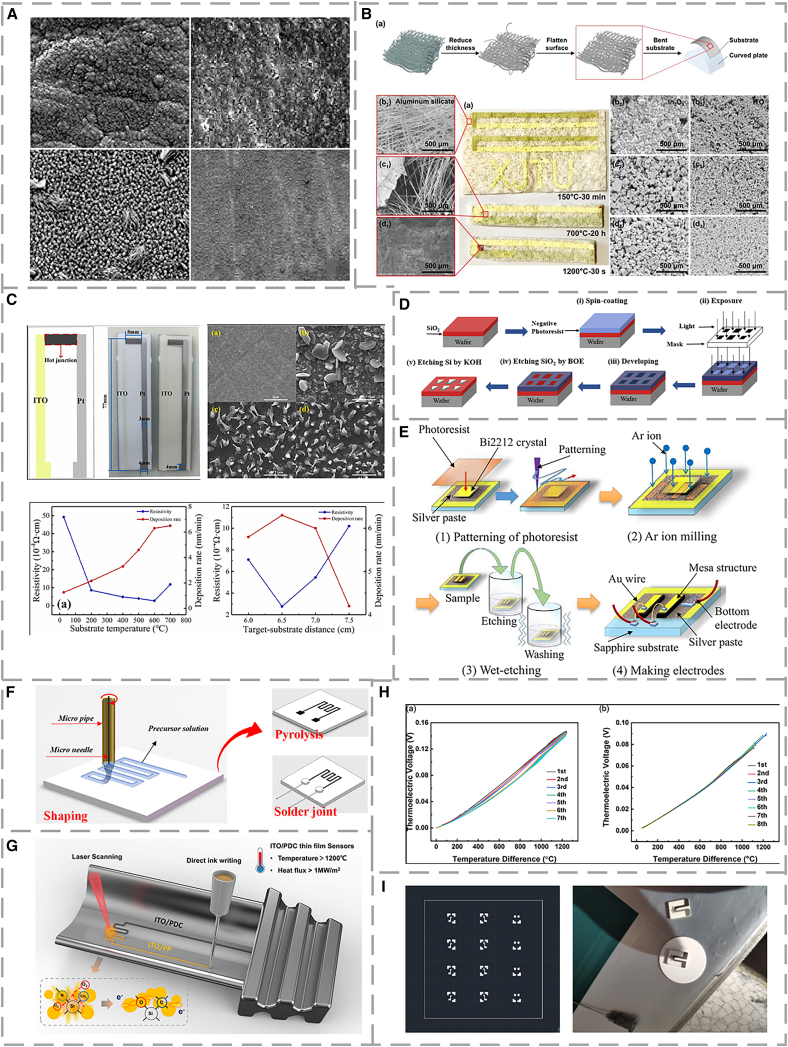
Advanced fabrication processes for HT-TFTCs (A) SEM micrographs of four distinct substrates. Reprinted from Zhang et al.,^106^ with the permission of IEEE. (B) Fabrication process and microstructure of aerogel pads. Reprinted from Liu et al.,^107^ with the permission of IOP Publishing. (C) Nanostack structures formed by PLD. Reprinted from Liu et al.,^108^ with the permission of Elsevier Publishing. (D) Procedural flow of the photolithography process. Reprinted from Lv et al.,^109^ with the permission of Frontiers Publishing. (E) Flowchart of the etching process. Reprinted from Shibano et al.,^110^ with the permission of AIP Publishing. (F) DIW fabrication process. Reprinted from Wu et al.,^111^ with the permission of Elsevier Publishing. (G) Direct write-laser composite technology. Reprinted from Xu et al.,^112^ with the permission of Elsevier Publishing. (H) Thermal cycling performance of ITO-In_2_O_3_ HT-TFTCs. Reprinted from Xu et al.,^113^ with the permission of Elsevier Publishing. (I) Configuration of the screen-printing mesh and the resulting HT-TFTC products. Reprinted from Wang et al.,^114^ with the permission of MDPI Publishing.

To build an HT-TFTC, first an appropriate substrate has to be selected, which acts as a structural carrier and fundamentally determines the performance and longevity of the final device.^101^^,^^102^^,^^103^^,^^104^^,^^105^ Zhang et al. systematically compared four types of ceramic substrates (Al_2_O_3_, SiO_2_, ZrO_2_, and SiC) for tungsten-rhenium (W-Re) thin films as illustrated in Figure 5A.^106^ SiC was identified as the best substrate as it resulted in the smoothest, finest grain size, and most dense films. Moreover, polymers such as polyimide (PI), polydimethylsiloxane (PDMS), and polyethylene terephthalate (PET) are becoming popular flexible substrates for film deposition on complicated surfaces.^78^^,^^115^^,^^116^^,^^117^^,^^118^^,^^119^^,^^120^^,^^121^^,^^122^^,^^123^^,^^124^^,^^125^ However, the lower thermal stability of conventional polymers limited their applicability. Thus, advanced substrates such as nanofiber mats and mica were developed.^75^^,^^78^^,^^107^^,^^126^^,^^127^ Liu et al. utilized an aerogel pad substrate, as exhibited in Figure 5B. Following thickness reduction and surface planarization to minimize roughness, the HT-TFTC showed excellent thermometric performance in the temperature range of −190°C to 1,200°C. Interestingly, the sensor managed to survive the 100 *g* impact acceleration and 2,000 Hz high-frequency vibration.

The deposition strategy is a key factor that intrinsically governs the microstructure and chemistry of thin films, and hence the final properties. As the dominating technique for physical vapor deposition (PVD), magnetron sputtering makes it possible to fine-tune electrical resistivity, interfacial adhesion, and thermoelectric stability by parameter optimization.^47^^,^^50^^,^^51^^,^^64^^,^^76^^,^^93^^,^^96^^,^^128^ Most recent studies have focused on using orthogonal experimental designs to obtain optimal sputtering parameters.^129^^,^^130^^,^^131^^,^^132^ In comparison, chemical vapor deposition (CVD) receives less attention, although it has better adhesion but lower deposition rates and is only used for barrier insulation layers.^133^^,^^134^ Meanwhile, pulsed laser deposition (PLD) is excellent for producing oxide-based materials (e.g., ITO, In_2_O_3_, and indium zinc oxide (IZO)). As seen in Figure 5C, Liu et al. utilized PLD to deposit ITO at a substrate temperature of 600°C, yielding a nanocone morphology with a low resistivity of 2.76 × 10^−4^ Ω cm. This configuration exhibited an average Seebeck coefficient exceeding 50 μV °C^−1^ (from room temperature to 1,300°C), short-term tolerance up to 1,400°C, and a rapid response time of 435 μs.^108^

Despite the maturity of PVD technology, its inherent “line-of-sight” propagation results in “shadowing effects” on complex curved surfaces, which can lead to significant thickness non-uniformity or even incomplete coverage.^135^^,^^136^^,^^137^^,^^138^^,^^139^ Consequently, this can lead to the occurrence of substantial thickness non-uniformity or even missing coating altogether. There are currently some methods available to mitigate this effect. For instance, tilted rotary sputtering with planetary substrate rotation can significantly improve thickness uniformity on curved surfaces such as aero-engine blades.^135^ Plasma-assisted deposition enhances surface diffusion and re-sputtering through high-energy ion bombardment, effectively suppressing voids in shadowed regions while improving film density and adhesion. Additionally, hybrid processes combining magnetron sputtering with adaptive direct ink writing (DIW), integrated with real-time thickness monitoring and feedback control, enable maskless conformal deposition on complex 3D geometries.^140^ These techniques provide practical pathways for the integration of HT-TFTCs onto curved components in aero-engines and other high-temperature applications.

After the uniform films are deposited on the surface of substrates, certain processes are performed to convert these films into sensor circuit structures. The major steps involved in the patterning process are photolithography and etching. The intricate pattern designed on the mask is precisely replicated on the photoresist over the substrate through exposure and development in photolithography. This is better explained in Figure 5D.^109^^,^^141^^,^^142^ The etching process uses chemical corrosion or physical bombardment to remove substrate from areas that are not protected by the photoresist permanently, as shown in Figure 5E.^110^^,^^111^^,^^143^^,^^144^ Although these methods yield high pattern fidelity, they require very flat substrates and cumbersome processes. Therefore, the direct production of sensors on three-dimensional curved objects, such as an aircraft engine blade, is impractical. In comparison, direct-writing and additive-manufacturing techniques are maskless digital processes that permit creation by layer-by-layer deposition.

The new direct writing and three-dimensional (3D) printing processes based on additive manufacturing principles enable quick, maskless, and digital prototyping. The said methods improve fabrication productivity and allow precise control of complex curvilinear surfaces. As shown in Figure 5F, DIW enables the conformal deposition of high-viscosity ceramic precursors on substrates for the construction of pre-designed microstructures with a minimum line width of 45 μm and thickness of approximately 2.7 μm.^140^^,^^145^ This technology is not just about the optimization of process flow; it also establishes the basis of HT-TFTCs for *in situ* integration with hot-section components. Such sensors exhibit long-term stability in 800°C oxidation with a resistance drift of 0.1% h^−1^.^60^

The “direct write-laser composite” technology combines the design flexibility of DIW with the high efficiency of laser processing. It therefore reduces the manufacturing cycle from hours, seen in photolithography, to minutes. As seen in Figure 5G, laser-induced transient heating facilitates the transformation of the pre-ceramic polymer into an amorphous ITO/PDC composite, while ITO particles are sintered to promote the formation of continuous conductive pathways to improve the performance of practical devices.^12^^,^^112^ Meanwhile, optimal inkjet printing enables the low-cost and atmospheric fabrication of high-performance HT-TFTCs by adjusting the ink composition and process conditions. The benefits, such as the low-temperature film formation, design flexibility, enhanced thermoelectric conversion efficiency, and high-temperature stability (able to withstand 1,100°C with less than 3% resistance change), allow for industrial scalability.^59^^,^^83^^,^^146^^,^^147^ The game-changing potential of these patterning techniques lies in their capacity to circumvent the two-dimensional limitations of conventional lithography, enabling the possibility for customization, small-scale production, and conformal integration.

While emerging patterning technologies are delivering game-changing solutions for stacking of curved surfaces, there are formidable barriers to their adoption as a replacement for conventional means for HT-TFTCs. One of the major limitations is resolution, which means that the size of features is limited by the size of nozzles or droplets. According to the study, direct writing and transfer printing usually achieve linewidths between 50 and 400 μm but are far away from the nano- or sub-micron capabilities of conventional photolithography.^148^^,^^149^ Furthermore, ceramic precursor inks have a rheological quandary. They must have low viscosity to allow for smooth extrusion, but they also need to solidify quickly to retain their shape. Thus, optimally formulating the inks is not so easy.^150^ Consequently, the advancement of these technologies hinges on overcoming processing bottlenecks and developing innovative functional inks. Post-deposition heat treatment is a critical process for modulating crystallinity, interfacial configuration, and elemental distribution—parameters that are decisive for the performance and stability of thermoelectric materials.^151^^,^^152^^,^^153^^,^^154^ Precise tuning of annealing parameters improves the overall properties of the thin-film elements considerably. Results from comparative studies between N_2_-air and vacuum-air annealing protocols confirm optimization of the linear response and high-temperature stability of ITO-based thermocouples using N_2_-air treatment. This atmospheric condition suppresses Sn segregation, thus lowering the resistivity drift rate from 0.5% to 0.375% h^−1^ and enhancing the thermal limit to 1,200°C.^155^^,^^156^

A two-step annealing strategy, which includes pre-annealing in a nitrogen atmosphere to induce N-doping followed by high-temperature air annealing, has enabled the ITO-In_2_O_3_ thermoelectric material to demonstrate superior thermal cycling reproducibility at 1,300°C. As illustrated in Figure 5H, the thermal cycling curves obtained from this method are closely aligned with minimal variation, showcasing exceptional reproducibility and high-temperature stability.^113^ These findings highlight the critical importance of annealing parameters in microstructural evolution, leading to a shift toward precision-regulated heat treatment technologies. The synergistic combination of screen printing and high-temperature sintering, utilizing a composite paste consisting of a “functional phase/glass phase/organic carrier,” results in thin films characterized by high density, strong adhesion, and superior oxidation resistance, as illustrated in Figure 5I.^114^ Although the resolution is generally constrained to line widths exceeding 50 μm due to mesh parameters and paste rheology, the simplicity and cost-effectiveness of this method make it highly suitable for mass production.^49^^,^^52^^,^^157^ Recently, laser pyrolysis has emerged as an advanced technique that modifies traditional reaction pathways to enhance graphitization and crystallization. This method facilitates the construction of a multiphase conductive network within the composite film, leading to a 67% reduction in resistivity compared to conventional furnace pyrolysis.^158^ The integration of screen printing with novel pyrolysis methods presents a scalable solution that effectively balances performance and economic efficiency for high-temperature sensor fabrication.^159^

The existing sensor manufacturing processes have reached a relative level of maturity. However, achieving ideal high-performance sensors necessitates stringent standards for critical metrics such as film thickness uniformity, patterning precision, and three-dimensional surface coverage. Each preparation method is inherently constrained by its underlying physicochemical mechanisms, which limits its performance across all parameters. Consequently, an isolated improvement strategy that focuses on refining a single process—such as optimizing sputtering parameters, innovating patterning methods, or enhancing thermal treatment schemes—will struggle to resolve these mutually constraining technical conflicts. Furthermore, the current processes do not adequately address the compound demands of future applications, which include enhanced spatial resolution, multifunctional component integration, and improved performance stability. The key to achieving technological breakthroughs lies in developing synergistic innovation models for hybrid processes, for instance, integrating the uniformity advantages of sputtering technology with the adaptability of direct-write techniques, or combining the precision characteristics of lithography with the efficiency features of screen printing. This approach facilitates the construction of customized, optimal fabrication systems tailored to diverse application requirements.

## Failure mechanisms and protection technologies for HT-TFTCs

**Figure 6:**
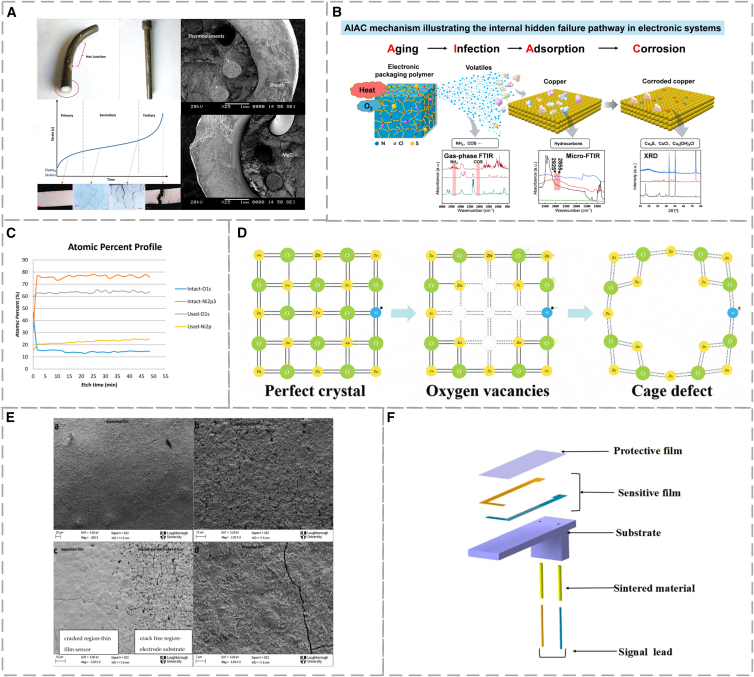
Failure modes and protection technologies (A) Corrosion-creep failure mode. Reprinted from Rakshan et al.,^160^ with the permission of Elsevier Publishing. (B) AIAC (aging-infection-adsorption-corrosion) mechanism illustrating the internal hidden failure pathway. Reprinted from Rakshan et al.,^161^ with the permission of Elsevier Publishing. (C) Atomic percentage profiles of Ni and O before and after the experiment. Reprinted from Guk et al.,^162^ with the permission of MDPI Publishing. (D) Schematic diagram of defect formation mechanisms. Reprinted from Luo et al.,^163^ with the permission of RSC Publishing. (E) Cracks appeared on the film. Reprinted from Guk et al.,^162^ with the permission of MDPI Publishing. (F) A lead-embedded connection solution. Reprinted from E et al.,^164^ with the permission of IOP Publishing.

In temperature monitoring within chemical process reactors, type K thermocouples experience accuracy degradation or complete failure after approximately 2 years of service at 400°C in sulfur/chlorine-containing atmospheres. This degradation arises from composite failure mechanisms, including creep-induced vacancy damage, grain boundary oxidation, and corrosive gas erosion as shown in Figure 6A.^160^^,^^165^ Research indicates that polymers within electronic devices release volatile species during thermal-oxidative aging. These evolved substances engage in an “aging-infection-adsorption-corrosion” (AIAC), as illustrated in Figure 6B. In this process, aging-induced gases migrate to and adsorb onto adjacent metal surfaces (e.g., Cu), triggering electrochemical reactions that produce corrosion products such as cuprous sulfide (Cu_2_S) or cuprous chloride (CuCl). Consequently, the electrical resistance of the metal conductors increases significantly, potentially leading to functional failure.^161^ These diverse failure modes and application scenarios demonstrate that ensuring long-term sensor stability necessitates an in-depth analysis of failure mechanisms and the establishment of corresponding protective systems. The inherently fragile nature of thin-film structures, combined with complex environmental factors such as high-temperature oxidation and periodic thermal shocks, contributes to material performance degradation, contact interface failure, and wire breakage. Addressing the limited lifespan of HT-TFTCs requires focused research into their core failure mechanisms, followed by the development of specialized protective solutions. This research is crucial for advancing technology from laboratory validation to practical engineering applications.

The degradation of materials performance and the loss of functionality are the most common and critical failure modes at high temperature. Thermocouple thin films react with oxygen in oxidizing conditions, which results in oxide layer formation. As shown in Figure 6C, the atomic percentage of oxygen of an 800°C thermally cycled Ni-based thermocouple across the entire 500 nm thickness shows oxidation extending to this depth of the film.^162^ To mitigate this issue, employing materials with excellent oxidation resistance is essential. Liu et al. chose In_2_O_3_ and ITO as thermocouple materials because of their robust oxidation resistance and Seebeck coefficient.^166^ They effectively suppress oxidation and volatilization by accurately controlling the annealing process parameters. The other efficient approach relies on the construction of the high-temperature, unprotective insulation systems.^103^^,^^167^^,^^168^^,^^169^^,^^170^^,^^171^ Lin et al. pioneered the use of amorphous AlON materials in high-temperature insulation systems.^172^ They developed a composite unprotective structure comprising “thermally grown Al_2_O_3_/amorphous AlON/vacuum-deposited Al_2_O_3_.” The high-temperature insulation performance of thin films utilizing this composite insulating layer is improved by nearly three orders of magnitude. Additionally, the layered film designed by Guo et al.^173^ incorporated a SiO_2_/Al_2_O_3_ composite thermal barrier layer alongside an Al_2_O_3_ protective layer. The Al_2_O_3_/Si_3_N_4_/YAlO sandwich-structured protective layer developed by Liu et al. demonstrated outstanding high-temperature oxidation resistance and thermodynamic stability.^174^

With regard to micro-mechanisms, Ruan et al. carried out a quantitative analysis on the distribution of oxidation spots.^175^ The authors revealed that horizontal oxidation diffusion is a key contributor to the sensor’s performance decline. Following this discovery, a full protection layer architecture and MEMS process improvement program was developed, which considerably enhanced the high-temperature reliability and service life of the device. According to Luo et al., the defect engineering mechanism is at atomic scale as presented in Figure 6D. Magnetron sputtering-deposited zinc-rich columnar Al-doped ZnO films dominate carrier transport by aluminum (Al), while cage-like defects effectively trap oxygen vacancies and help avoid migration instability during high-temperature operation.^163^ The invention of protective materials not only represents a fundamental breakthrough but also enhances the intrinsic stability. The future protective technology will keep a distance from its single-layer model to a “matrix-functional layer-composite protection” strategy. This method seeks to achieve deep atomic-level defect regulation and interface engineering to suppress oxidation diffusion behavior.

Mechanical constraints in ultra-thin films are predominantly characterized by heat-induced micro-fractures. Figure 6E illustrates prominent transverse and longitudinal micro-cracks that develop after thermal cycling (20°C–800°C), which arise from the significant thermal stress induced by the CTE mismatch between the Ni-based film and the substrate.^162^ Consequently, when the accumulated stress exceeds the interfacial bonding force, the films may experience peeling, cracking, or substantial delamination. Zhang et al. conducted a “three-factor, three-level” orthogonal test aimed at mitigating thermal stress-induced delamination failure in W-Re thermocouple films.^69^ The analysis of variance identified temperature as the most critical factor, followed by substrate thickness, while film thickness exhibited the least influence. The optimized configuration involved a 100-μm-thick SiC substrate paired with a 1-μm-thick W-3Re film, which achieved the lowest thermal stress under 600°C operating conditions. Furthermore, Pang et al. designed a three-layer packaging system composed of glass glaze, high-temperature ceramic adhesive, and alumina. This system effectively addresses issues of cracking, permeation, and thermal mismatch defects that are prevalent in conventional single- or double-layer packaging by coordinating thermal expansion coefficients and enhancing both high-temperature stability and mechanical strength.^176^

Furthermore, welded or crimped joints between HT-TFTCs and external wires are susceptible to oxidation, poor contact, or film separation at elevated temperatures, representing a common weak link in the measurement circuit. Consequently, Cui et al. developed an integrated architecture for leads and sensor substrates.^177^ This innovative approach fundamentally transformed the traditional “planar film/external lead” design paradigm by converting external leads into embedded structural units, thereby facilitating seamless integration with the insulating substrate. By reconfiguring the sensor’s physical structure and integration methods, the authors successfully eliminated this critical failure point at the lead junction. Additionally, Research Group E introduced a lead-embedded connection solution utilizing Al_2_O_3_ ceramic substrates, as depicted in Figure 6F.^164^ This group circumvented connection failures associated with silver-paste bonding or soldering by employing high-temperature sintering to achieve metallurgical bonding between leads and substrates. These groundbreaking structural optimization strategies effectively address long-standing technical challenges that impact measurement reliability at the system integration level.

To conclude, the issues surrounding the performance degradation and protection of thermally stable thin-film sensors are still major challenges. As increasing demands for extreme-temperature environments subject existing materials to extreme maximums of thermal resistance, reliability remains the Achilles’ heel of the new technological sensors among them. It is particularly important to study new high-temperature material systems and make use of different technical approaches to fortify structural stability.

## Self-generation and self-repair technology in HT-TFTCs

Frequent thermal shocks in aircraft engines and hypersonic vehicle engines lead to the formation of microcracks in thermocouples.^31^^,^^178^ Prolonged stretching or bending of flexible wearable electronics can result in cracks within conductive films.^179^^,^^180^^,^^181^ Consequently, moving beyond traditional passive protection, self-healing technology imparts intelligent regenerative properties to sensors, thereby facilitating long-term applications. The autonomous whisker growth and self-healing mechanisms for damage in HT-TFTCs have emerged as a significant area of research. This technology enables instantaneous repair of thermocouples upon sustaining damage, thereby significantly extending their service life.

**Figure 7:**
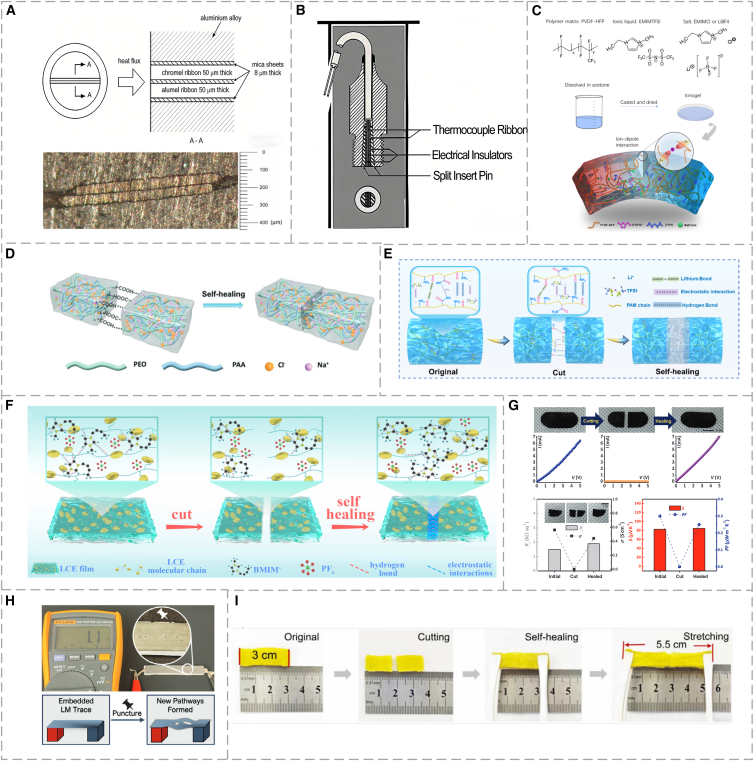
Self-healing and self-renewing mechanisms of HT-TFTCs (A) The eroded thermocouple. Reprinted from Wanga et al.,^184^ with the permission of SAE Publishing. (B) Schematic representation of a self-renewing thermocouple. Reprinted from Wanga et al.,^185^ with the permission of SPIE Publishing. (C) Synthetic route of the ionogel and illustration of the corresponding ion-dipole interactions. Reprinted from Liu et al.,^186^ with the permission of Elsevier Publishing. (D) Schematic of the self-healing process in PAA-PEO-NaCl ionic hydrogels. Reprinted from Fu et al.,^187^ with the permission of Wiley Publishing. (E) Mechanism of the self-healing process mediated by reversible interactions. Reprinted from Dai et al.,^188^ with the permission of Elsevier Publishing. (F) Schematic diagram of the self-healing mechanism of Liquid Crystal Elastomer (LCE) i-TE film. Reprinted from Cao et al.,^189^ with the permission of Elsevier Publishing. (G) Comparison of the performance of SIS/P3BT/tris(pentafluorophenyl)borane (BCF) composite films before and after self-healing. Reprinted from Jeong et al.,^190^ with the permission of Wiley Publishing. (H) Digital photograph of a thermosensitive elastomer (TE) specimen punctured by a pushpin (top) and the corresponding mechanism of electrical self-healing (bottom). Reprinted from Han et al.,^191^ with the permission of Wiley Publishing. (I) Self-healing image of cationic high-entropy gel (CHEG). Reprinted from Yang et al.,^192^ with the permission of Wiley Publishing.

A practical approach to restoring sensor functionality involves reconstructing failed functional junctions. Early researchers employed mechanical force intervention in related studies. Jedrzejowski et al. pioneered wear-based thermocouple measurement technology.^182^^,^^183^ As illustrated in Figure 7A, this technique utilizes a coaxial probe structure and grinding materials for on-site processing of the probe tip. Essentially, this technology represents a specialized method for directly forming thermoelectric junctions at measurement points through mechanical abrasion.^184^^,^^193^^,^^194^ However, simulation studies conducted by Buttsworth et al. indicated that insulation performance declines after 3–5 wear-regeneration cycles due to constraints imposed by the characteristics of the insulating layer,^195^ reflecting limitations in the cyclic stability of this renewable technology.^196^ Furthermore, each thermocouple requires manual polishing, which is inefficient and complicates the determination of optimal polishing timing for operators. In contrast, Nanigian et al. successfully developed the “self-renewing thermocouple,” as shown in Figure 7B.^185^ During polishing, softer metallic materials (e.g., copper-nickel alloys) generate micrometer-scale metallic whiskers. These whiskers penetrate the insulating layer and form connections with adjacent metal strips, creating hundreds of parallel micro-junctions. When surface wear occurs, newly exposed metallic layers automatically form fresh thermoelectric junctions.^197^^,^^198^^,^^199^^,^^200^^,^^201^^,^^202^^,^^203^

Mechanical regeneration utilizes physical methods to achieve structural recovery and offers universal applicability independent of material chemical properties. However, this approach is limited by cumulative damage and a finite repair lifespan, making non-destructive or automated instantaneous repair difficult to realize. Despite being in a nascent stage, emerging thermocouple technology based on surface erosion principles is regarded as a promising direction. In contrast, intrinsic self-healing materials mimic biological mechanisms to repair damage via intelligent chemical strategies. Key mechanisms involve interfacial reorganization based on reversible bonds, elasticity-assisted reconstruction of conductive pathways, and the shape memory effect.^204^^,^^205^^,^^206^^,^^207^^,^^208^^,^^209^ Given the limitations of shape memory polymers at high temperatures, current research primarily focuses on the first two mechanisms. This classification establishes a theoretical basis for developing intelligent sensing materials tailored to specific temperature ranges.

Among various self-healing materials, ion gels have emerged as a prominent research focus for flexible, low-to-medium-temperature thermoelectric sensing due to their unique ion-transport mechanisms and dynamically reversible crosslinking properties, resulting in significant advancements in recent years. Liu et al. developed an ionic gel that integrates exceptional ductility, self-healing capabilities, excellent light transmittance, and an adjustable p- or n-type high thermoelectric potential (Figure 7C).^186^ The self-healing property of the gel arises from the reversible ion-dipole interactions formed between polyvinylidene fluoride-hexafluoropropylene (PVDF-HFP) and ionic liquid/salt. Concurrently, Fu et al. constructed a system based on a polyacrylic acid-polyethylene oxide (PAA-PEO) physical crosslinking network (Figure 7D).^187^ Additionally, Liu et al. successfully fabricated a high-efficiency thermal battery utilizing polyvinyl alcohol (PVA) hydrogel.^210^ Moreover, Dai et al. developed polyacrylamide (PAM)/LiTFSI_4_ ionic thermoelectric hydrogel fibers exhibiting outstanding self-healing properties (Figure 7E).^188^ Cao et al. created a room-temperature self-healing n-type liquid-crystal elastomer thermoelectric ionic gel (Figure 7F).^189^ All the materials achieve self-healing through noncovalent interactions, including dynamic metal coordination bonds and reversible interionic hydrogen bonds.^211^^,^^212^^,^^213^ These technological breakthroughs provide new insights for the development of flexible thermoelectric devices that integrate stretchability, transparency, and self-healing properties.

Despite allowing driving force-induced strong intrinsic molecular self-healing at low temperatures, the use of chemical repair strategies is subject to the thermal stability of dynamic reversible bonds. When the thermal kinetic energy becomes larger than the bond energy, elevated temperatures will cause the interfacial and bulk crosslinked networks to dissociate. The occurrence of this phenomenon prevents organized structural reorganization and also causes material degradation (e.g., softening) before the activation of repair takes place. Thus, such materials cannot be used in an operating environment above 300°C.^214^^,^^215^

In addition to chemical repair methods, the use of physical interactions that can reconstruct conductive pathways is an effective strategy. This method offers distinct advantages for regaining electrical functionality.^216^^,^^217^ Such methods typically make use of the flow properties of liquid metals and the movement of polymer segments to reconnect the broken regions. Jeong et al. developed an all-organic self-healing thermoelectric material.^190^ As shown in Figure 7G, through the rearrangement of the molecular chains of the styrene-isoprene-styrene block copolymer (SIS) elastomer and re-establishment of the poly(3-butylthiophene-2,5-diyl) (P3HT) nanowire conductive network, self-healing is achieved. After being repaired, the morphology is restored, as well as thermoelectric conversion efficiency and extensibility. Han et al. designed a 3D flexible self-healing electrode system incorporating liquid metal, as shown in Figure 7H.^191^ Because of the liquid nature of the material, it automatically re-injects into the damaged region, restoring effective electrical connectivity using a Ga-In eutectic alloy. Furthermore, the gallium oxide coating created on the surface of the metal speeds up circuit recovery when contact occurs at fracture interfaces for quick electrical self-healing. Meanwhile, Yang et al. successfully synthesized a cationic high-entropy gel exhibiting exceptional self-healing properties, as shown in Figure 7I.^192^ Its high entropy reduces the barriers to molecular chain motion, enabling damage repair within just 10 min at room temperature without external intervention. Test data revealed that the repaired material achieved a 98% recovery rate in elongation at break and retained 82% of its tensile strength, confirming its highly efficient self-healing performance. Such self-healing material systems are based on physical mechanisms, particularly those that incorporate composite designs combining liquid metals with dynamic polymer networks. These systems provide effective solutions for the rapid restoration of material functionality, especially electrical conductivity.

Physical repair strategies for HT-TFTCs are constrained by material property evolution in extreme environments. Oxidation and degradation processes significantly compromise metal fluidity and polymer molecular chain mobility. For instance, gallium-based liquid metals rapidly form a solid Ga_2_O_3_ shell at elevated temperatures, which inhibits flow and prevents effective micro-crack filling.^218^ Similarly, acrylamide polymers suffer from chain scission and structural collapse.^219^ Furthermore, oxidation-induced compositional changes promote particle agglomeration and disrupt matrix homogeneity, ultimately leading to the complete failure of the self-healing mechanism.

In recent years, new references on high-temperature self-healing ceramic coatings remain limited, and such material systems are still relatively rare. This scarcity represents a major bottleneck in the field. Therefore, although the flexible materials introduced earlier cannot operate under the extreme high-temperature conditions (>800 °C) focused on in this review, their self-healing mechanisms (such as reversible bonds and liquid metal flow) provide fundamental inspiration for the design of future high-temperature self-healing systems.

The NANMAC (a brand/supplier of thermocouples and temperature sensors) self-regenerating thermocouple and the NASA ceramic-based HT-TFTCs exemplify the two most engineering-viable self-healing paradigms in the current landscape of high-temperature sensing. The NANMAC system has achieved 3 to 5 *in situ* thermoelectric junction reconstructions in extreme transient environments such as rocket nozzles, solid boosters, and nuclear fusion devices by utilizing an erosion-driven active metal whisker regeneration mechanism. This technology has reduced sensor replacement frequency by over 70% and has achieved a thermoelectric output recovery rate exceeding 90%, establishing itself as the standard “multi-use disposable” thermometry solution for major programs including NASA, SpaceX, and ITER. Conversely, the Pt13Rh/Pt ceramic HT-TFTC developed by the NASA Glenn Research Center employs an Al_2_O_3_/YSZ self-healing protective coating to facilitate oxidative filling and whisker-like bridging of microcracks during thermal cycles at 1,500°C–1,700°C. This mechanism has extended the continuous service life to between 50 and 1,000 h with a thermal drift of less than 2°C per hour, directly supporting the life cycle assessment of hot-section components for the space shuttle, Rolls-Royce Spey engines, and subsequent hypersonic propulsion systems. Collectively, these cases validate that self-repair strategies based on damage-induced whisker/oxide bridging have transitioned from laboratory concepts to aerospace engineering practices.

## Discussion

### Development overview of HT-TFTCs

Conventional thermocouples incorporate wired technology with a protective covering. Industrially, thermocouple types, like K, S, and B, are quite popular and well established. A comparative analysis of conventional thermocouples and HT-TFTCs in dynamic measurement and microscale applications is presented. While they have limitations, they offer significant potential advantages. Traditional sensors with a large thermal mass and macroscopic size (1 mm scale) have long response latencies in the 0.5–5 s range. On the contrary, HT-TFTCs achieve an order-of-magnitude improvement in response time using the very low thermal inertia afforded by micro- and nanofabrication, reaching response times in the millisecond or even microsecond range (e.g., 8.4 μs for ITO/In_2_O_3_ films). This can accurately capture transient heat fluctuations in aero-engine combustion chambers.

Regarding structural intrusiveness, the physical bulkiness of traditional probes will disturb the natural topology and flow fields of the part being tested. Unlike traditional corrugated surfaces, the two-phase flow turbine cascades with their micron profiles allow for conformal *in situ* deposition on curvilinear blades with little interference with aerodynamic flows or structural stress distributions. Conventional noble-metal thermocouples, such as types S and R, are remarkably durable at high temperatures. Nevertheless, their performance is limited by their relatively low Seebeck coefficients, which are approximately 10 μV °C^−1^. In contrast, newer ceramic-based films (e.g., NiCoCrAlY/Al_2_O_3_) outperform standard thermocouples by over 2 orders of magnitude, reaching coefficients of 375 μV °C^−1^, thus enhancing signal strength. Nevertheless, thin-film technology generally has good long-term stability. In extreme environments, state-of-the-art thermocouples offer a service life of several years, unlike thin-film devices, which are susceptible to oxidation at high temperatures, elemental volatilization, and interfacial delamination. As a result, their present application is mainly restricted to short-term precision diagnosis, which may lie in hour’s scale and few hundred hours.

Beyond these individual performance metrics, a fundamental design dilemma in HT-TFTCs lies in the intrinsic trade-off between sensitivity, response time, and high-temperature stability. As summarized in Table 1, achieving microsecond-level response times often requires ultra-thin film architectures, which may compromise thermoelectric output and accelerate thermal degradation. Conversely, optimizing sensitivity through increased thickness or specific material compositions can elevate thermal inertia, prolonging response latency. This multidimensional coupling necessitates a balanced design strategy tailored to specific application scenarios, rather than the pursuit of any single performance extreme.

**Table 1:** Development overview of HT-TFTCs

Thin-film material	Preparation method	Seebeck coefficient	Maximum working temperature	Response time	Application scenario	Reference
ITO (N-doped): ITO (O-doped)	sputtering	6 μV °C^−1^	1,200°C	–	gas turbine engine temperature monitoring	Gregory et al.^57^
ITO (N-doped): NiCoCrAlY/alumina nanocomposite	sputtering	375 μV °C^−1^	1,200°C	–		Gregory et al.^57^
La_0_._8_Sr_0_._2_CrO_3_	RF sputtering	167.8 μV °C^−1^	1,100°C	–	high-temperature measurement in aero-engines	Liu et al.^154^
In_1_._35_ZnO_2_._11_/In_2_O_3_	screen printing	64.9 μV °C^−1^	1,100°C	–		Zhang et al.^52^
AZO (Al-doped ZnO), ITO/AZO	–	168 μV °C^−1^	500°C	–	thermocouple	Luo et al.^163^
ITO/In_2_O_3_	RF magnetron sputtering	129.26 μV °C^−1^	1,450°C	8.4 μs	high-temperature transient temperature measurement	Xie et al.^50^
ITO/Pt	RF magnetron sputtering	76.1 μV °C^−1^	900°C	–		Liu et al.^46^
In_2_O_3_/Pt	RF magnetron sputtering	203.9 μV °C^−1^	900°C	–	high-temperature transient temperature measurement	Liu et al.^46^
ITO/In_2_O_3_	RF magnetron sputtering	138.1 μV °C^−1^	1,200°C	–		Liu et al.^46^
Ni/Ca_3_Co_4_O_9_	RF magnetron sputtering	153 μV °C^−1^ (positive electrode), 912 μV °C^−1^ (series 6)	105°C (test)	–	thin-film thermocouple, energy harvesting	Xin et al.^43^
Pt/ITO	pulsed laser deposition	50 μV °C^−1^	1,400°C (short term)	435 μs	surface measurement of high-temperature components (aero-engines)	Liu et al.^108^
Pt-Rh10/Pt	screen printing	10.70 μV °C^−1^	1,500°C	535 μs	dynamic temperature test for micro detonator ignition	Wang et al.^114^
ITO/In_2_O_3_	inkjet printing	111 μV °C^−1^	800°C	–	aerospace, steel metallurgy	Lei et al.^83^
In_2_O_3_ (YSZ/Al_2_O_3_ doped)/In_2_O_3_	screen printing	180.9 μV °C^−1^	1,850°C	2.8 ms	ultra-high-temperature measurement (aero-engines)	Wang et al.^44^
ITO/In_2_O_3_ with HfO_2_	magnetron sputtering	–	1,100°C	–	high spatial resolution of high-temperature field in extreme environments	Shao et al.^170^
ITO/In_2_O_3_ with microcrystalline glass	magnetron sputtering, tape mask	–	1,100°C (130 h)	–	turbine guide vane of an aero-engine	Zhao et al.^103^

As illustrated in Table 1, wire-type sensors perform one order of magnitude worse than HT-TFTCs. This signifies a major shift in technology from bulk metal alloys to micro- and nanoscale functional coatings. There is a marked difference in the behavior and sensitivity. Types K, S, and B thermocouples are based on a standardized mechanical wire-drawing process. The response times of said materials are typically 1–5 s, indicating that they have substantial thermal inertia. However, oxide ceramics (like ITO and In_2_O_3_) in thin-film technologies can give a leap progression, with a microsecond reaction time (e.g., 8.4 μs ITO/In_2_O_3_). Additionally, the Seebeck coefficient of thin-film materials can reach about 375 μV °C^−1^; in other words, it is nearly 10 times higher than that of platinum-rhodium sensors (10 μV °C^−1^). High-temperature sensors are relatively mature and stable with very high temperatures, 1,820°C for type B conventional sensors. New fabrication methods like RF magnetron sputtering and PLD show promise for non-invasive conformal integration. This structural transformation is an answer to the aerodynamic disturbance of the sensors. Thin-film devices are extremely significant for measuring transient thermal shocks in extreme aerospace propulsion scenarios.

Beyond these individual performance metrics, a fundamental design dilemma in HT-TFTCs lies in the intrinsic trade-off between sensitivity, response time, and high-temperature stability. As summarized in Table 1, achieving microsecond-level response times often requires ultra-thin-film architectures, which may compromise thermoelectric output and accelerate thermal degradation. Conversely, optimizing sensitivity through increased thickness or specific material compositions can elevate thermal inertia, prolonging response latency. This multidimensional coupling necessitates a balanced design strategy tailored to specific application scenarios, rather than the pursuit of any single performance extreme.

### Current key challenges in HT-TFTCs

Although significant progress has been made in each dimension, HT-TFTCs still face several critical challenges in practical engineering applications. These challenges are summarized in Table 2 across the five dimensions.

**Table 2:** Current key challenges and future directions

Dimension	Current major challenges	Future directions
Materials	difficulty in simultaneously achieving high-temperature stability and high thermoelectric conversion efficiency; elemental volatilization and unstable oxygen vacancies	multi-component composite doping and atomic-scale defect engineering; development of novel high-entropy ceramic materials
Structural design	complex multi-layer structures, high risk of thermal stress mismatch and interfacial delamination	simplified structures combined with biomimetic hierarchical interfaces; AI-assisted topology optimization
Fabrication processes	poor conformal deposition uniformity on curved surfaces and insufficient process reproducibility	hybrid fabrication processes (sputtering and direct writing); intelligent digital monitoring systems
Failure protection	insufficient long-term service life (mostly less than 500 h); difficulty in completely suppressing oxidation diffusion	multi-layer composite protective coatings and atomic-scale defect engineering; service life prediction models
Self-healing technology	scarcity of high-temperature self-healing material systems (few effective systems above 800°C); limited repair cycles	digital twin-driven intelligent self-healing systems; high-temperature reversible dynamic covalent bond materials; external field (thermal/light/electrical) triggered self-repair

Table 2 summarizes the primary challenges currently faced by each dimension along with corresponding future directions. Particularly noteworthy is the potential of AI-assisted design and digital twin technology as key tools for overcoming existing bottlenecks. For example, machine-learning-based multi-objective optimization can simultaneously balance thermoelectric performance, structural stress distribution, and process parameters. Digital twin models can simulate sensor degradation behavior in extreme environments in real time and predict optimal self-healing activation timing, thereby enabling on-demand intelligent repair. These forward-looking technologies are expected to drive HT-TFTCs from passive protection toward active intelligent regeneration, providing long-life, high-reliability temperature monitoring solutions for advanced equipment such as aero-engines and hypersonic vehicles.

### Multidimensional coupling and collaborative design

While individual advancements in materials, structural design, fabrication processes, failure protection, and self-healing technologies have significantly pushed the performance boundaries of HT-TFTCs, the interactions and trade-offs among these five dimensions remain underexplored in most existing literature. A true breakthrough toward long-life, high-reliability HT-TFTCs requires systematic multidimensional collaborative design rather than isolated optimization.

Material selection directly influences process compatibility and structural integrity. For instance, ceramic oxide films (e.g., ITO/In_2_O_3_ or YSZ-doped In_2_O_3_) exhibit superior high-temperature stability and oxidation resistance compared to noble metals, yet they often suffer from poorer conformal deposition uniformity on complex curved surfaces due to higher brittleness and sensitivity to shadowing effects during PVD. In contrast, metallic films (e.g., Pt/PtRh or W-Re) offer better ductility and adhesion on curved aero-engine blades but are more prone to oxidation and elemental diffusion at temperatures above 1,000°C. Structural designs, such as the sandwich architecture with NiCrAlY bond layer and Al_2_O_3_ thermally grown oxide, effectively mitigate thermal stress mismatch and reduce oxidation diffusion pathways by providing a dense, gradual transition from metal to ceramic layers. However, increased layering complexity can compromise fabrication precision and introduce new interfacial delamination risks under thermal cycling.

Fabrication processes further couple with failure mechanisms: magnetron sputtering ensures excellent thickness control and thermoelectric properties for most materials, yet its line-of-sight limitation exacerbates non-uniformity on 3D geometries, accelerating localized oxidation and mechanical cracking in unprotected regions. Self-healing technologies introduce additional trade-offs. Mechanical self-renewing approaches (e.g., erosion-driven whisker regeneration in NANMAC-type sensors) or oxide-bridging protective coatings extend service life through *in situ* junction reconstruction, but they may slightly degrade baseline Seebeck coefficient or introduce contact resistance variations after multiple repair cycles. Intrinsic chemical self-healing (e.g., ion gels or liquid metal composites) excels at low-to-medium temperatures but risks thermal decomposition or reduced carrier mobility above 800°C, potentially lowering overall thermoelectric conversion efficiency.

To quantify these couplings, orthogonal experimental designs and response surface methodologies have proven effective for multi-factor optimization. Our team previously conducted a three-factor, three-level orthogonal experiment on W-Re thin-film thermocouples (TFTCs) to analyze thermal stress as a function of film thickness, substrate thickness, and temperature, identifying temperature as the dominant factor and optimizing configurations to minimize delamination. Similar approaches have been applied to deposition parameters and embedded sensors in cutting tools.^220^ However, comprehensive studies integrating all five dimensions—material doping, sandwich structures, hybrid fabrication, composite protection, and self-healing—remain scarce. Emerging machine-learning-assisted multi-objective optimization, such as Convolutional Neural Network – Long Short-Term Memory network (CNN-LSTM) error correction in doped In_2_O_3_ systems, offers promising pathways to address these complex trade-offs.^221^^,^^222^

### Comprehensive performance evaluation standards and reliability assessment

Recent advancements in material doping and structural optimization have significantly enhanced the potential of HT-TFTCs. However, a rigorous and standardized evaluation system is essential to bridge the gap between laboratory prototypes and industrial applications. Therefore, we propose a multi-dimensional assessment framework. It encompasses static sensitivity, dynamic transient response, high-temperature reliability mechanisms, and statistical engineering consistency. This framework quantitatively validates the effectiveness of optimized designs. Key performance indicators should include temperature range and accuracy, and response time. Material standards must ensure high purity,^223^^,^^224^^,^^225^^,^^226^^,^^227^ corrosion resistance life,^226^^,^^228^ assess vibration resistance, and so on.^229^^,^^230^^,^^231^^,^^232^^,^^233^^,^^234^ Additionally, safety specifications must encompass failure analysis, system integration compatibility, and environmental compliance. The adoption of hierarchical systems and international testing methods will ensure reliable application in extreme high-temperature environments.

## Conclusions

HT-TFTC technology has achieved significant breakthroughs. Its application scope has expanded from laboratory settings to engineering practices, demonstrating exceptional advantages in precise and high-efficiency temperature detection under extreme conditions. This review systematically summarizes progress across five core dimensions: material systems, structural design, fabrication processes, failure mechanisms with protection, and self-healing technologies. It identifies that promoting multi-dimensional synergistic design and fusion innovation is critical. In particular, the vigorous development of self-healing technologies with “intelligent regeneration” capabilities represents a crucial pathway to overcoming existing performance bottlenecks and realizing long-life, highly reliable, and intelligent applications. The further development of this technology is anticipated to resolve testing challenges associated with critical components in high-end equipment, such as engines and aircraft. This advancement will provide essential technical support for the design and testing of new aerospace equipment models that feature higher speeds and improved thrust-to-weight ratios.

## Acknowledgments

This study was supported by the 10.13039/501100001809National Natural Science Foundation of China (no. 52475570), the S&T Program of Energy Shaanxi Laboratory (no. ESLB202437), the 10.13039/501100015401Key Research and Development Program of Shaanxi (no. 2025CY-YBXM-053), and the 2025 Shaanxi Province “Sanqin Bochuang” Talent Support Program (2025SQBC001).

## Author contributions

Zhang.Z.: writing – review and editing and conceptualization; Zhang T. and Liu Z.: writing – review and editing; Wang M.: methodology; Zhou G. and Liu X.: methodology; Wu C. and Fang X.: methodology; Duan D. and Tian B.: methodology.

## Declaration of interests

The authors declare no competing interests.

## References

[1] Kou Z., Wang Q., Li G., Zhang W. (2023). Application of thermocouple temperature measurement technology for aero-engine high-temperature wall. J. Eng. Therm. Energy Power.

[2] Sun D., Cui Z., Zhou Y., Zhou P., Yi W., Lin X., Wang L. (2018). Electro-Mech. Eng.

[3] Powers S.W., Schetz J.A., Lowe K.T., Kapania R.K. (2021). Analysis of stresses in metal sheathed thermocouples in high-temperature flows. AIAA J..

[4] Wrbanek J., Fralick G., Farmer S., Sayir A., Blaha C., Gonzalez J., Tanaka M. (2004). 40th AIAA/ASME/SAE/ASEE Joint Propulsion Conference and Exhibit.

[5] Von Moll A., Behbahani A.R., Fralick G.C., Wrbanek J.D., Hunter G.W. (2014). 50th AIAA/ASME/SAE/ASEE Joint Propulsion Conference.

[6] Zhao C., Zhou G., Zhang J., Zhang Z., Huang G., Xie Q., Zhao C., Zhou G., Zhang J., Zhang Z. (2025). Dynamic error correction for fine-wire thermocouples based on CRBM-DBN with PINN constraint. Symmetry.

[7] Omega E. (2024). Omega Complete Temperature Measurement Handbook and Encyclopedia MMV.

[8] Liu D., Shi P., Ren W., Liu Y., Niu G., Liu M., Zhang N., Tian B., Jing W., Jiang Z., Ye Z.G. (2018). A new kind of thermocouple made of p-type and n-type semi-conductive oxides with giant thermoelectric voltage for high temperature sensing. J. Mater. Chem. C.

[9] Chopra K.L., Bahl S.K., Randlett M.R. (1968). Thermopower in thin-film copper—constantan couples. J. Appl. Phys..

[10] Delatorre R.G., Sartorelli M.L., Schervenski A.Q., Pasa A.A., Güths S. (2003). Thermoelectric properties of electrodeposited CuNi alloys on Si. J. Appl. Phys..

[11] Matus L.G. (2013). Instrumentation for aerospace applications: electronic-based technologies. J. Aero. Eng..

[12] Xu L., Zhou X., Huang Y., Wang Y., Shao C., Li Y., Wang L., Yang Q., Sun D., Chen Q. (2024). Design and fabrication of metal spherical conformal thin film multisensor for high-temperature environment. Chin. J. Aeronaut..

[13] Duan F.L., Xie Z., Ji Z., Weng H. (2020). Robust thin-film temperature sensors embedded on nozzle guide vane surface. AIAA J..

[14] Guo H., Liu Z., Guo T., Sun Y., Shen K., Wang B., Cheng Y., Wang Y., Ma T., Wang Z., Ding W. (2024). Effect of hot junction size on the temperature measurement of proton exchange membrane fuel cells using NiCr/NiSi thin-film thermocouple sensors. Micromachines.

[15] Miyamoto A., Lee S., Cooray N.F., Lee S., Mori M., Matsuhisa N., Jin H., Yoda L., Yokota T., Itoh A. (2017). Inflammation-free, gas-permeable, lightweight, stretchable on-skin electronics with nanomeshes. Nat. Nanotechnol..

[16] Guimarães B., Fernandes C.M., Figueiredo D., Carvalho O., Miranda G., Silva F.S. (2024). Multi-material laser powder bed fusion of embedded thermocouples in WC-Co cutting tools. J. Manuf. Process..

[17] Chen J., Lin Y., Zhao D., Gao S., Zheng M., Ma W., Chen B. (2024). Integrated design and fabricate of high sensitivity built-in thin-film thermocouple temperature measurement tool. J. Mech..

[18] Martin L.C., Holanda R., Minnifield N.K. (1994). NASA/SPIE Conference on Spin-off Technologies from NASA for Commercial Sensors and Scientific Applications (SPIE).

[19] Duan F.L., Li J., Gao J., Ding G., Cao X. (2018). Integrated fabrication of high-temperature microelectromechanical system sensor on aeroengine turbine blade. J. Thermophys. Heat Tran..

[20] Grassini S., Fulginiti D., Pisano R., Oddone I., Parvis M. (2014). 2014 IEEE International Symposium on Medical Measurements and Applications (memea).

[21] Batine A., Boumegnane A., Nadi A., Cochrane C., Cherkaoui O., Tahiri M. (2025). Advances in screen-printed conductive inks for sustainable and high-performance E-textiles: innovations, challenges, and future prospects. Adv. Eng. Mater..

[22] Martins B., Patacas C., Cavaleiro A., Faia P., Fernandes F. (2025). Real-time temperature monitoring during titanium alloy machining with cutting tools integrating novel thin-film sensors. Mech. Syst. Signal Process..

[23] Wang Y., Sun Y., Lei D., Xue Y. (2023). Thermal oxidation reliability and structure optimization of thin film thermocouples. J. Beijing Univ. Aeronaut. Astronaut..

[24] Wang M., Wang Z., Han B., Li Y., Ge X. (2016). High temperature measurement technology for main combustor of aeroengine. Aeroengine.

[25] Han B., Wang M., Li Y., Ma Z. (2017). Application of gas analysis method in exit temperature field test of high temperature rise full annular combustor. Aeroengine.

[26] Martin L.C., Fralick G.C., Taylor K.F. (1999). Advances in Thin Film Thermocouple Durability under High Temperature and Pressure Testing Conditions.

[27] Wrbanek J., Fralick G., Blaha C., Busfield A., Thomas V. (2002). 38th AIAA/ASME/SAE/ASEE Joint Propulsion Conference & Exhibit.

[28] Wrbanek J.D., Fralick G.C. (2006).

[29] Wrbanek J.D., Fralick G.C., Gonzalez J.M., Laster K.L.. (2008). Thin Film Ceramic Strain Sensor Development for High Temperature Environments.

[30] Renn M.J., Schrandt M., Renn J., Feng J.Q. (2017). Localized laser sintering of metal nanoparticle inks printed with aerosol jet® technology for flexible electronics. J. Microelectron. Electron. Packag..

[31] Ruan Y., Li J., Xiao Q., Wu Y., Shi M. (2023). High-temperature failure evolution analysis of K-type film thermocouples. Micromachines.

[32] Zhao X., Liang X., Jiang S., Zhang W., Jiang H., Zhao X., Liang X., Jiang S., Zhang W., Jiang H. (2017). Microstructure evolution and thermoelectric property of Pt-PtRh thin film thermocouples. Crystals.

[33] Burns G.W., Ripple D.C., Battuello M. (1998). Platinum versus palladium thermocouples: an emf-temperature reference function for the range 0 °C to 1500 °C. Metrologia.

[34] Moiseeva N.P. (2004). The prospects for developing standard thermocouples of pure metals. Meas. Tech..

[35] Hill K.D. (2002). An investigation of palladium oxidation in the platinum/palladium thermocouple system. Metrologia.

[36] Abdelaziz Y.A., Megahed F.M., Halawa M.M. (2004). Stability and calibration of platinum/palladium thermocouples following heat treatment. Measurement.

[37] Ahmed M.G., Ali K. (2008). Investigating Pt/pd thermocouples in the temperature range from 800 °C to 1500 °C at the national institute of standards NIS-egypt. MAPAN-J. Metrol. Soc. India.

[38] Ma K., Cao L., Luo F., Zhou H., Liu D., Luo B., Xu Y., Cui J., Zhao X. (2022). Highly oriented platinum/iridium thin films for high-temperature thermocouples with superior precision. Phys. Chem. Chem. Phys..

[39] Kreider K.G., Gillen G. (2000). High temperature materials for thin-film thermocouples on silicon wafers. Thin Solid Films.

[40] Wrbanek J.D., Fralick G.C., Zhu D. (2012). Ceramic thin film thermocouples for SiC-based ceramic matrix composites. Thin Solid Films.

[41] Gregory O.J., Amani M., Tougas I.M., Drehman A.J. (2012). Stability and microstructure of indium tin oxynitride thin films. J. Am. Ceram. Soc..

[42] Gregory O.J., You T., Crisman E.E. (2005). Effect of aluminum doping on the high-temperature stability and piezoresistive response of indium tin oxide strain sensors. Thin Solid Films.

[43] Xin B., Paul B., le Febvrier A., Eklund P. (2023). Thin-film thermocouples of Ni-joined thermoelectric Ca3Co4O9. Mater. Sci. Semicond. Process..

[44] Wang M., Zhang Z., Lei J., Li L., Li B., Liu Z., Xia Y., Liu D., Tian B., Jing W. (2025). Performance evaluation and correction of Al2O3 and YSZ-doped In_2_O3/In2O3 multilayer heterogeneous thin-film thermocouples up to 1850 °C. J. Adv. Ceram..

[45] Tougas I., Amani M., Gregory O. (2013). Metallic and ceramic thin film thermocouples for gas turbine engines. Sensors.

[46] Liu Y., Shi P., Ren W., Huang R., Liu Y., Shi P., Ren W., Huang R. (2023). Thermoelectrical properties of ITO/Pt, In2O3/Pt and ITO/In2O3 thermocouples prepared with magnetron sputtering. Crystals.

[47] Liu Y., Lin T., Huang R., Shi J., Chen S. (2024). Analysis of the effect of copper doping on the optoelectronic properties of indium oxide thin films and the thermoelectric properties of an In2O3/Pt thermocouple. Crystals.

[48] Liu J., Tian B., Lu N., Liu Z., Zhang Z., Shi M., Fang X., Feng K., Tan Q., Liu D. (2024). Study on aluminium oxide doping modification of indium oxide and thermoelectric properties. Ceram. Int..

[49] Zhang Z., Tian B., Li L., Lei J., Liu Z., Liu J., Cheng G., Zhao N., Fang X., Zhao L. (2022). Thermoelectricity and antivibration properties of screen-printed nanodoped In1.35ZnO2.11/In2O3 thin-film thermocouples on alumina substrates. Ceram. Int..

[50] Xie S., Jiang H., Zhao X., Deng X. (2023). Fabrication and performances of high-temperature transient response ITO/In2O3 thin-film thermocouples. J. Mater. Sci. Mater. Electron..

[51] Zhang Z., Li S., Tian B., Liu Z., Liu J., Cheng G., Fan X., Fang X., Zhao N., Zhao L. (2022). Simulation, fabrication, and characteristics of high-temperature, quick-response tungsten–rhenium thin-film thermocouples probe sensor. Meas. Sci. Technol..

[52] Zhang Z., Liu J., Cai R., Liu Z., Lei J., Sun R., Wu N., Zhao N., Tian B., Zhao L. (2022). High-temperature-sensing smart bolt based on indium tin oxide/In2O3 thin-film thermocouples with nickel-based single-crystal superalloy via screen printing. Chemosensors.

[53] Kreider K.G., DiMeo F. (1998). Platinum/palladium thin-film thermocouples for temperature measurements on silicon wafers. Inside NIST.

[54] Zhao X., Wang R., Liu Y., Deng X., Jiang H., Zhang W. (2020). High temperature thermoelectric properties of nitrogen doped ITO thin films. Vacuum.

[55] Fan X., Tian B., Zhang Z., Shi M., Zhao K., Zhou G., Liu Z., Lei J., Wang M., Li S. (2025). Indium oxide doped with zirconium oxide thin film thermocouple with a temperature upper limit of 1600 °C. Ceram. Int..

[56] Liu F., Guo Y., Zhang M. (2022). Characteristic analysis of IWO conductive thin films with different doping ratios. Electron. World.

[57] Gregory O.J., Busch E., Fralick G.C., Chen X. (2010). Preparation and characterization of ceramic thin film thermocouples. Thin Solid Films.

[58] Li Y., Xu Y., Sun D., Chen Q., Fang L., Hai Z., Cui Z., Li X., He G., Cui J. (2021). SiBCN high temperature thin film temperature sensor. Micronanoelectron. Technol..

[59] He G., He Y., Xu L., Li L., Wang L., Hai Z., Sun D. (2023). La(Ca)CrO3-filled SiCN precursor thin film temperature sensor capable to measure up to 1100 °C high temperature. Micromachines.

[60] Chen Q., Zhang P., Liu K., Xu P., Wei H., Hai Z., Wu D., Zhao Y., Jin X., Wang X., Sun D. (2023). Polymer-derived ceramic thin-film thermocouples for high temperature measurements. Ceram. Int..

[61] Yang Q., Chen P., Li X., Zhu Y. (2024). Rational design of SiBCN ceramics with excellent attenuation to strong electromagnetic-wave-absorbing properties at low frequency. Composites, Part B.

[62] Niu J., Meng S., Jin H., Li J., Yi F., Zhou Y. (2019). Thermal stability and nanostructure evolution of amorphous SiCN ceramics during laser ablation in an argon atmosphere. J. Eur. Ceram. Soc..

[63] Xu R. (2022). Master’s thesis, Dalian Univ. Technol..

[64] Wang B., Liu X., Peng B., Li L., Yue H., Fang S., Zhang W. (2020). Pt/ITO thin film electrode SAW temperature sensor resistant to 1000°C high temperature. China Meas. Test.

[65] Gu B., Zhao Z., Chen H., Pan W., Zhang Z. (2020). Development of thermocouple sensor based on Ni-based alloy thin film. Opt. Optoelectron. Technol..

[66] Liu Z., Tian B., Zhang B., Liu J., Zhang Z., Wang S., Luo Y., Zhao L., Shi P., Lin Q., Jiang Z. (2021). A thin-film temperature sensor based on a flexible electrode and substrate. Microsyst. Nanoeng..

[67] Zhao X., Li H., Chen Y., Jiang H. (2017). Preparation and thermoelectric characteristics of ITO/Pt thin film thermocouples on Ni-based superalloy substrate. Vacuum.

[68] Fan X., Tian B., Shi M., Zhang Z., Liu Z., Zhou G., Liu J., Li L., Lin Q., Jiang Z. (2024). Sandwich probe temperature sensor based on In2O3-IZO thin film for ultra-high temperatures. Int. J. Extrem. Manuf..

[69] Zhang Z., Tian B., Yu Q., Shi P., Lin Q., Zhao N., Jing W., Jiang Z. (2017). Range analysis of thermal stress and optimal design for tungsten-rhenium thin film thermocouples based on ceramic substrates. Sensors.

[70] Liu Z., Wang Q., Guo S., Wang H., Jiang W., Liu S., Liu C., Wang N., Cui Y., Ding W. (2023). The preliminary exploration on change mechanism of seebeck coefficient for NiCr/NiSi thin film thermocouple with different thickness. J. Alloys Compd..

[71] Luo B., Cao L., Ma K., Zhang Z., Luo F., Zhou H., Liu D., Xu Y., Cui J., Zhao X. (2022). Hierarchical iridium nanostructure-based thin films with high-temperature stability and oxidation resistance for thermocouples. ACS Appl. Nano Mater..

[72] Tian B., Liu Z., Wang C., Liu Y., Zhang Z., Lin Q., Jiang Z. (2020). Flexible four-point conjugate thin film thermocouples with high reliability and sensitivity. Rev. Sci. Instrum..

[73] Li P., Yang Y., Chen J., Zhao L., Ren T. (2025). Highly sensitive spider-slit-organ-inspired crack-based flexible temperature sensor. RSC Adv..

[74] Ren H., Li W., Li H., Ding Y., Li J., Feng Y., Su Z., Zhang X., Jiang L., Liu H., Hu P. (2025). Jellyfish-inspired high-sensitivity pressure-temperature sensor. Adv. Funct. Mater..

[75] Liu Z., Tian B., Li Y., Lei J., Zhang Z., Liu J., Lin Q., Lee C., Jiang Z. (2023). A large-area bionic skin for high-temperature energy harvesting applications. Nano Res..

[76] Shuai H., Dai Y., Jiang H. (2025). Development of transient K-type thermocouple probe. Meas. Control Technol.

[77] Yang K., Jiang H., Zhao X. (2019). Development of thin film thermocouple for aeroengine. Transducer Microsyst. Technol..

[78] Zhang Z., Liu Z., Lei J., Chen L., Li L., Zhao N., Fang X., Ruan Y., Tian B., Zhao L. (2023). Flexible thin film thermocouples: from structure, material, fabrication to application. iScience.

[79] Lei J.-F., Will H.A. (1998). Thin-film thermocouples and strain-gauge technologies for engine applications. Sens. Actuators, A.

[80] Tougas I.M., Gregory O.J. (2013). 2013 IEEE SENSORS.

[81] Liu Z., Yang Y., Sun Y., Guo T., Shen K., Cheng Y., Wang B., Hu K., Zhang C., Hao G. (2025). Measurement and analysis of surface temperature gradients on magnetron sputtered thin film growth studied using NiCr/NiSi thin film thermocouples. Small.

[82] Yuan Y., Najafi K. (2019). Vertical self-defined thin-film thermoelectric thermocouples by angled Co-evaporation for use in μTEGs. J. Phys., Conf. Ser..

[83] Lei J., Tian B., Liu X., Wang M., Li L., Liu Z., Liu J., Zhang Z., Shi M., Tan Q., Qi R. (2025). High temperature resistant thin film thermocouple prepared based on inkjet printing. Ceram. Int..

[84] Kim D.M., Kwak H.J., Shin D.Y., Park J.H., Kim J.Y. (2024). Optimal fabrication of a thin-film thermocouple (TFTC) using alumel/chromel junctions. Heliyon.

[85] Liu H., Sun W., Chen Q., Xu S. (2011). Thin-film thermocouple array for time-resolved local temperature mapping. IEEE Electron Device Lett..

[86] Liu Z., Tian B., Liu B., Liu X., Lei J., Liu J., Zhang Z., Wu C., Lin Q., Jiang Z. (2025). High-stability flexible thin-film temperature sensor using MWCNTs-toughened peano structure. Microsyst. Nanoeng..

[87] Dong H., Lu M., Wang W., Tan Q. (2024). High temperature heat flux sensor with ITO/In2O3 thermopile for extreme environment sensing. Microsyst. Nanoeng..

[88] Li J., Tao B., Huang S., Yin Z. (2019). Cutting tools embedded with thin film thermocouples vertically to the rake face for temperature measurement. Sens. Actuators, A.

[89] Tian X., Kennedy F.E., Deacutis J.J., Henning A.K. (1992). The development and use of thin film thermocouples for contact temperature measurement. Tribol. Trans..

[90] Scarioni L., Castro E.M. (2000). Thermoelectric power in thin film Fe–CuNi alloy (type-J) couples. J. Appl. Phys..

[91] Chen Y.Z., Jiang H.C., Zhang W.L., Liu X.Z., Jiang S.W. (2013). Film thickness influences on the thermoelectric properties of NiCr/NiSi thin film thermocouples. Mod. Phys. Lett. B.

[92] Luo B., Cao L., Luo F., Zhou H., Ma K., Liu D., Wang L., Hu S., Sun K., Zhang S. (2022). Highly ordered columnar ITO thin film with enhanced thermoelectric and mechanical performance over wide temperature range. Ceram. Int..

[93] Luo B., Cao L., Gao H., Zhang Z., Luo F., Zhou H., Ma K., Liu D., Miao M. (2022). Superior thermoelectric performance of robust column-layer ITO thin films tuning by profuse interfaces. ACS Appl. Mater. Interfaces.

[94] He M., Tian B., Zhang Z., Fan X., Liu Z., Chen L., Fang X. (2025). High-precision measurement of high temperature in dual-junction thin-film thermocouples under small temperature gradients. IEEE Trans. Instrum. Meas..

[95] Tian B., Xing Y., Zhang X., Liu Z., Zhang Z., Liu J., Zhang B., Lin Q., Jiang Z. (2022). A high-precision three-dimensional probe array temperature sensor. Chemosensors.

[96] Tian B., Liu J., Zhang Z., Liu Z., Ma R., Lin Q., Jiang Z. (2024). Research on high-precision thin-film temperature sensor based on multi-node array structure. J. Mech. Eng..

[97] Zhao Y., Feng H., Lou W., Li L., Wang Q., Ding G., Zhang C. (2025). Intelligent temperature measuring thermal spray multilayer thermal barrier coatings based on embedded thin film thermocouples. J. Colloid Interface Sci..

[98] Hao S., Fu Q., Meng L., Xu F., Yang J. (2022). A biomimetic laminated strategy enabled strain-interference free and durable flexible thermistor electronics. Nat. Commun..

[99] Tougas I.M., Gregory O.J. (2013). Thin film platinum–palladium thermocouples for gas turbine engine applications. Thin Solid Films.

[100] Seo J., Lee S., Sohn I., Cho M., Park S., Yang W., Lee C., Kang Y., Yang K., Koo B. (2025). Unveiling substrate effects: nanoscale insights into Sb2Te3 thin films grown by atomic layer deposition. Surf. Interfaces.

[101] Wang H. (2025). Research on NiCr/NiSi thin film thermocouple sensor for measuring the surface temperature of automobile engine. Front. Mater..

[102] Tian B., Zhang Z., Shi P., Zheng C., Yu Q., Jing W., Jiang Z. (2017). Tungsten-rhenium thin film thermocouples for SiC-based ceramic matrix composites. Rev. Sci. Instrum..

[103] Zhao F., Zhang P., Qiang D., Shao C., Jia S., Zhu K., Chen S., Sun D., Chen Q. (2025). Thin-film thermocouple with microcrystalline glass encapsulation for high-temperature applications. IEEE Sens. J..

[104] El-Hamid H.K.A., Gaber A.A., Ngida R.E.A., Sadek H.E.H., Khattab R.M., Mandour H.S. (2024). Study of microstructure and corrosion behavior of nano-Al2O3 coating layers on TiO2 substrate via polymeric method and microwave combustion. Sci. Rep..

[105] Liu Y., Ren W., Shi P., Liu D., Zhang Y., Liu M., Ye Z.-G., Jing W., Tian B., Jiang Z. (2018). A highly thermostable In2O3/ITO thin film thermocouple prepared via screen printing for high temperature measurements. Sensors.

[106] Zhang Z., Tian B., Du Z., Yu Q., Lin Q., Zhao N., Jiang Z. (2018). 2018 IEEE 13th Annual International Conference on Nano/micro Engineered and Molecular Systems (NEMS).

[107] Liu Z., Tian B., Jiang Z., Li S., Lei J., Zhang Z., Liu J., Shi P., Lin Q. (2022). Flexible temperature sensor with high sensitivity ranging from liquid nitrogen temperature to 1200 °C. Int. J. Extrem. Manuf..

[108] Liu T., Dong H., Wang H., Niu Y., Li X., Zhang L., Xiong J., Tan Q. (2023). Nano cone ITO thin films prepared by pulsed laser deposition for surface measurement of high-temperature components. J. Alloys Compd..

[109] Assumpcao D., Kumar S., Narasimhan V., Lee J., Choo H. (2018). High-performance flexible metal-on-silicon thermocouple. Sci. Rep..

[110] Yang S.M., Chung L.A. (2021). A thermoelectric energy generator with double cavity design by single polysilicon layer in standard CMOS process. IEEE Sens. J..

[111] Shibano Y., Kashiwagi T., Komori Y., Sakamoto K., Tanabe Y., Yamamoto T., Minami H., Klemm R.A., Kadowaki K. (2019). High-Tc superconducting THz emitters fabricated by wet etching. AIP Adv..

[112] Sun D., Hai Z., Zhao F., Fu Y., Tang L., Zeng Y., Chen G., Wu C., Wang L., Chen Q. (2024). Rapid laser fabrication of indium tin oxide and polymer-derived ceramic composite thin films for high-temperature sensors. J. Colloid Interface Sci..

[113] Lv Z., Zhang C., Wang Y., Kang Z., Gao X., Guo Y. (2023). Enhanced high-temperature stability of indium tin oxide - indium oxide thermocouples by two-step annealing. Thin Solid Films.

[114] Wang F., Lin Z., Zhang Z., Li Y., Chen H., Liu J., Li C. (2022). Fabrication and calibration of Pt-Rh10/Pt thin-film thermocouple. Micromachines.

[115] Zhang F., Zang Y., Huang D., Di C.a., Zhu D. (2015). Flexible and self-powered temperature–pressure dual-parameter sensors using microstructure-frame-supported organic thermoelectric materials. Nat. Commun..

[116] Konishi S., Hirata A. (2019). Flexible temperature sensor integrated with soft pneumatic microactuators for functional microfingers. Sci. Rep..

[117] Rahman M.T., Cheng C.-Y., Karagoz B., Renn M., Schrandt M., Gellman A., Panat R. (2019). High performance flexible temperature sensors via nanoparticle printing. ACS Appl. Nano Mater..

[118] Lee S.H., Shen H., Han S. (2019). Flexible thermoelectric module using Bi-Te and Sb-Te thin films for temperature sensors. J. Electron. Mater..

[119] Seo B., Hwang H., Kang S., Cha Y., Choi W. (2018). Flexible-detachable dual-output sensors of fluid temperature and dynamics based on structural design of thermoelectric materials. Nano Energy.

[120] Wang Q., Wang Y., Chen L. (2019). A green composite hydrogel based on cellulose and clay as efficient absorbent of colored organic effluent. Carbohydr. Polym..

[121] Liu H., Sun W., Xu S. (2012). An extremely simple thermocouple made of a single layer of metal. Adv. Mater..

[122] Jeon J.G., Kim H.J., Shin G., Han Y., Kim J.H., Lee J.H., Lee J., Lim H., Ha S., Bae M. (2022). High-precision ionic thermocouples fabricated using potassium ferri/ferrocyanide and iron perchlorate. Adv. Electron. Mater..

[123] Xin Y., Zhou J., Lubineau G. (2019). A highly stretchable strain-insensitive temperature sensor exploits the seebeck effect in nanoparticle-based printed circuits. J. Mater. Chem. A.

[124] Chen Y., Lei H., Gao Z., Liu J., Zhang F., Wen Z., Sun X. (2022). Energy autonomous electronic skin with direct temperature-pressure perception. Nano Energy.

[125] Jung M., Lee J., Vishwanath S.K., Kwon O.-S., Ahn C.W., Shin K., Jeon S. (2020). Flexible multimodal sensor inspired by human skin based on hair-type flow, temperature, and pressure. Flexible Printed Electron..

[126] Han D., Li G., Zhou S., Wang Z., Yang F., Xu S. (2017). To save half contact pads in 2D mapping of local temperatures with a thermocouple array. RSC Adv..

[127] Liu Z., Tian B., Liu X., Zhang X., Li Y., Zhang Z., Liu J., Lin Q., Jiang Z. (2023). Multifunctional nanofiber mat for high temperature flexible sensors based on electrospinning. J. Alloys Compd..

[128] Zhang W., Shen T., Wang L., Zhao W., Deng Y. (2024). Integration process of Pt/PtRh high temperature thin film thermocouple sensor based on magnetron sputtering. J. Mater. Eng..

[129] Tian B., Yu Q., Zhang Z., Du Z., Ren W., Shi P., Jiang Z. (2018). Effect of magnetron sputtering parameters on adhesion properties of tungsten-rhenium thin film thermocouples. Ceram. Int..

[130] Ouyang L., Gao Y., Zheng H. (2024). Research on the improvement of the adhesion strength of the Cu films deposited on the Al2O3 films. J. Vac. Sci. Technol., B.

[131] Her S.-C., Chang C.-F. (2017). Fabrication and characterization of indium tin oxide films. J. Appl. Biomater. Funct. Mater..

[132] Zhang Y. (2020). Master's thesis, Shanghai Jiao Tong Univ.

[133] Selvakumar N., Barshilia H.C. (2012). Review of physical vapor deposited (PVD) spectrally selective coatings for mid- and high-temperature solar thermal applications. Sol. Energy Mater. Sol. Cell..

[134] Jones J.G., Jero P.D., Garrett P.H. (1998). In-situ control of chemical vapor deposition for fiber coating. Eng. Appl. Artif. Intell..

[135] Bishop N., Walker J., DeRoo C.T., Liu T., Tendulkar M., Cotroneo V., Hertz E.N., Kradinov V., Schwartz E.D., Reid P.B. (2019). Thickness distribution of sputtered films on curved substrates for adjustable x-ray optics. JATIS.

[136] Shen J., Lu L., Du C., Ran J., Min R., Yang F., Ma K. (2024). Preparation of TSV barrier layer by magnetron sputtering based on tilting and rotating method. Semicond. Tech.

[137] Haque S.M., Rao K.D., Misal J.S., Tokas R.B., Shinde D.D., Ramana J.V., Rai S., Sahoo N.K. (2015). Study of hafnium oxide thin films deposited by RF magnetron sputtering under glancing angle deposition at varying target to substrate distance. Appl. Surf. Sci..

[138] Liu H., Ma D., Li Y., You L., Leng Y., Liu H., Ma D., Li Y., You L., Leng Y. (2023). Evolution of the shadow effect with film thickness and substrate conductivity on a hemispherical workpiece during magnetron sputtering. Metals.

[139] Panjan P., Drnovšek A., Gselman P., Čekada M., Panjan M., Panjan P., Drnovšek A., Gselman P., Čekada M., Panjan M. (2020). Review of growth defects in thin films prepared by PVD techniques. Coatings.

[140] Zhang L., Kang L., Yu B., Ma C., Yang M., Jiang J., Han D., Wang H., Zhang R., Shao G. (2026). Conformal high-resolution printing of polymer-derived ceramics by self-adaptive direct ink writing. J. Eur. Ceram. Soc..

[141] Shinozuka J., Basti A., Obikawa T. (2008). Development of cutting tool with built-In thin film thermocouples for measuring high temperature fields in metal cutting processes. J. Manuf. Sci. Eng..

[142] Lv L., Liu T., Jiang T., Li J., Zhang J., Zhou Q., Dhakal R., Li X., Li Y., Yao Z. (2023). A highly sensitive flexible capacitive pressure sensor with hierarchical pyramid micro-structured PDMS-based dielectric layer for health monitoring. Front. Bioeng. Biotechnol..

[143] Datta A., Choi H., Li X. (2006). Batch fabrication and characterization of micro-thin-film thermocouples embedded in metal. J. Electrochem. Soc..

[144] Yang S.M., Wang S.H. (2021). Development of a thermoelectric energy generator with double cavity by standard CMOS process. IEEE Sens. J..

[145] Xu L., Zhou X., Zhao F., Fu Y., Tang L., Zeng Y., Chen G., Wu C., Wang L., Chen Q., Yang K. (2022). High-temperature electrical properties of polymer-derived ceramic SiBCN thin films fabricated by direct writing. Ceram. Int..

[146] Liu L., Zhang M., Chen X., Zhu J., Qiu L. (2025). Research on inkjet printing manufacturing method of high temperature thin film sensor for turbine blade. J. Aero. Power.

[147] Liu J., Xu L., Zhou X., Zhao F., Wang Y., Wang S., Lv W., Sun D., Chen Q. (2024). 3D-printed conformal thin film thermocouple arrays for distributed high-temperature measurements. Coatings.

[148] Zhuang Q., Zhang Y., Liu X., Xiao W., Chen Z., Lu L., Ding Z., Chen S., Chen Q., Patel S. (2025). Laser-assisted direct three-dimensional printing of free-standing thermoset devices. Nat. Electron..

[149] Kong X., Liu Y., Wang J., Yue L., Gong M., Lin X., Gao F., Zhang L., Wang D. (2025). Mask-free direct printing of highly customizable, conformable, robust, and recyclable microelectrodes for advanced curvy electronics. Small.

[150] Ponder J.F., Posey N.D., Germanton G., Advincula A.A., Zackasee J.L.S., Ramakrishnan S., Pruyn T.L., Dickerson M.B. (2026). Effects of corona size on the rheology and ceramic yield of preceramic polymer grafted nanoparticles. J. Am. Ceram. Soc..

[151] Jiao R., Wang K., Xin Y., Sun H., Gong J., Yu L., Wang Y. (2023). Enhancing the temperature coefficient of resistance of Pt thin film resistance-temperature-detector by short-time annealing. Ceram. Int..

[152] Liu D., Jiao R., Sun C., Wang Y., Liu D., Jiao R., Sun C., Wang Y. (2024). Effects of Substrates on the Performance of Pt Thin-Film Resistance Temperature Detectors. Coatings.

[153] Liu Z., Liang J., Zhou H., Lu W., Li J., Wang B., Li Q., Zhao X., Xu J. (2022). Stability enhancement of the nitrogen-doped ITO thin films at high temperatures using two-step mixed atmosphere annealing technique. Appl. Surf. Sci..

[154] Liu D., Shi P., Liu Y., Zhang Y., Tian B., Ren W., Liu D., Shi P., Liu Y., Zhang Y. (2021). Optimizing the Properties of La0.8Sr0.2CrO3 Thin Films through Post-Annealing for High-Temperature Sensing. Nanomaterials.

[155] Zhao X., Li H., Yang K., Jiang S., Jiang H., Zhang W. (2017). Annealing effects in ITO based ceramic thin film thermocouples. J. Alloys Compd..

[156] Kaźmierczak-Bałata A., Bodzenta J., Szperlich P., Jesionek M., Michalewicz A., Domanowska A., Mayandi J., Venkatachalapathy V., Kuznetsov A., Kaźmierczak-Bałata A. (2024). Impact of Annealing in Various Atmospheres on Characteristics of Tin-Doped Indium Oxide Layers towards Thermoelectric Applications. Materials.

[157] Duan S., Ren X., Zhang X., Cheng S., Hu W. (2018). Screen printed flexible electronic devices. Prog. Chem..

[158] Xu L., Cui Z., Li L., He Y., Wu C., Chen G., Li X., He G., Hai Z., Chen Q., Sun D. (2022). In situ laser fabrication of polymer-derived ceramic composite thin-film sensors for harsh environments. ACS Appl. Mater. Interfaces.

[159] Cui Z., Li X., Chen G., Wu C., He G., Hai Z., Chen Q., Sun D. (2022). Thin-film temperature sensor made from particle-filled polymer-derived ceramics pyrolyzed in vacuum. J. Eur. Ceram. Soc..

[160] Rakshan Kumar J.K., Bhattacharjee D., Dsilva P., Praveen R., Hegde S.R. (2023). Creep cavitation damage of K-type thermocouples. Eng. Fail. Anal..

[161] Shen K., Deng Y., Yang P., Liu Q., Lu T., Niu Y., Lv Y., Chen H., Li G. (2026). Failure from within: volatiles from polymer aging trigger electronic corrosion. Polym. Degrad. Stabil..

[162] Guk E., Ranaweera M., Venkatesan V., Kim J.-S., Guk E., Ranaweera M., Venkatesan V., Kim J.-S. (2016). Performance and durability of thin film thermocouple array on a porous electrode. Sensors.

[163] Luo B., Cao L., Zhang J., Luo F., Zhou H., Ma K., Beltrán-Pitarch B., Fuente M.S.D.l., Falomir F.V., García-Cañadas J. (2022). Defect governed zinc-rich columnar AZO thin film and contact interface for enhanced performance of thermocouples. Phys. Chem. Chem. Phys..

[164] E M., Cui Y., Guo S., Liu H., Ding W., Yin J. (2023). Novel lead-connection technology for thin-film temperature sensors with arbitrary electrode lengths. Meas. Sci. Technol..

[165] Zhang X. (2014). Improvement of flue gas temperature detection technology for Ausmelt furnace. China Nonferrous Metall..

[166] Liu Y., Ren W., Shi P., Liu D., Liu M., Jing W., Tian B., Ye Z., Jiang Z. (2017). Preparation and thermal volatility characteristics of In2O3/ITO thin film thermocouple by RF magnetron sputtering. AIP Adv..

[167] Liu Y., Jiang H., Zhao X., Deng X., Zhang W. (2022). High temperature electrical insulation and adhesion of nanocrystalline YSZ/Al2O3 composite film for thin-film thermocouples on Ni-based superalloy substrates. Appl. Surf. Sci..

[168] Li S., Zhang Z., Wang K., He M., Lei J., Li L., Tan Q., Qi R., Liu Z., Tian B. (2025). Enhanced temperature sensing performance of T-type TFTCs with SiC encapsulation layer from liquid nitrogen to 900 °C. Measurement.

[169] Sun N., Jiang H., Zhao X., Deng X., Zhang W. (2024). High-temperature protection performance of Mg-doped Al2O3 protective layers on the thin film thermocouples. Ceram. Int..

[170] Shao C., Fu Y., Qiang D., Zhang P., Tang L., Li Y., Hu L., Zeng Y., Huang G., Zhao F. (2025). High spatial resolution high-temperature thin-film temperature sensor with HfO2 protective layer. IEEE Sens. J..

[171] Ma J., Dong H., Miao Y., Wang Y., Jia Z., Tan Q. (2025). Enhanced high temperature thermal stability of In2O3-IHFO thin film thermocouples using composite protective layers. J. Eur. Ceram. Soc..

[172] Lin Z., Ma Y., Gu S., Wang W., Zhao X. (2021). Preparation and insulation performance of AlON/Al2O3 composite insulating layer on superalloy. Electron. Compon. Mater..

[173] Guo L., Zhang M., Jin Y., Wang Y., Hu G., Yang S., Zhang C. (2022). Development of a novel high temperature thin film heat flux sensor based on thermopile. Chin. J. Sensors Actuators.

[174] Liu Y., Jiang H., Zhao X., Liu B., Jia Z., Deng X., Zhang W. (2022). High temperature protection performance of sandwich structure Al2O3/Si3N4/YAlO multilayer films for Pt–Pt10%Rh thin film thermocouples. Ceram. Int..

[175] Ruan Y., Xue M., Teng J., Wu Y., Shi M. (2022). Horizontal oxidation diffusion behavior of MEMS-based tungsten-rhenium thin film thermocouples. Materials.

[176] Pang Y., Zhang C., Lei P., Huang M., Liang X. (2021). Research on packaging of platinum thin film resistance temperature sensor. Meas. Control Technol..

[177] Cui Y., Gao F., Zhu X., Su X., Yin J. (2020). Development and performance research of thin film temperature sensor for spacecraft. Acta Aeronautica Astronautica Sinica.

[178] Li Z., Tao B., Zhao R., Yang K., Chen X., Xie T., Xia Y., Zhu H., Tian H., Yu Y. (2024). Novel flexible atomic layer thermopile heat flux sensor via orientation-controlled growth technique. Ceram. Int..

[179] Chen K., Yuan Z., Dai S., Zhou J., Yu K. (2023). Preparation and performance of self-cleaning photothermal-induced self-healing flexible sensors. Compos. Sci. Technol..

[180] Tao Y.-K., Liu Y.-F., Dong J.-X. (2014). Flexible stop and double-cascaded stop to improve shock reliability of MEMS accelerometer. Microelectron. Reliab..

[181] Zhao Z., Fu H., Tang R., Zhang B., Chen Y., Jiang J. (2023). Failure mechanisms in flexible electronics. Int. J. Smart Nano Mater..

[182] Jedrzejowski S. (1998). Numerical processing of the eroding thermocouple signals related to heat transfer analysis In the reciprocating compressor. WIT Trans. Eng. Sci..

[183] Jedrzejowski S. (2000). Influence of material thermal properties on instantaneous heat flux measurements using an eroding thermocouple. WIT Trans. Eng. Sci..

[184] Wang X., Stone R., Stevens R., Arita Y., Buttsworth D. (2006).

[185] Nanigian J., Nanigian D., Korman V. (2006). Sensors for Propulsion Measurement Applications (SPIE).

[186] Liu S., Yang Y., Chen S., Zheng J., Lee D.G., Li D., Yang J., Huang B. (2022). High p- and n-type thermopowers in stretchable self-healing ionogels. Nano Energy.

[187] Fu M., Sun Z., Liu X., Huang Z., Luan G., Chen Y., Peng J., Yue K. (2023). Highly stretchable, resilient, adhesive, and self-healing ionic hydrogels for thermoelectric application. Adv. Funct. Mater..

[188] Dai Y., Wang H., Qi K., Ma X., Wang M., Ma Z., Wang Z., Yang Y., Ramakrishna S., Ou K. (2024). Electrode-dependent thermoelectric effect in ionic hydrogel fiber for self-powered sensing and low-grade heat harvesting. Chem. Eng. J..

[189] Cao L., Sun T., Zhao H., Wang L., Jiang W. (2025). Ultra-tough, self-healing, n-type liquid crystal elastomer thermoelectric lonogels via synergistic hydrogen-bonds and electrostatic interaction. Chem. Eng. J..

[190] Jeong Y.J., Jung J., Suh E.H., Yun D.-J., Oh J.G., Jang J. (2020). Self-healable and stretchable organic thermoelectric materials: electrically percolated polymer nanowires embedded in thermoplastic elastomer matrix. Adv. Funct. Mater..

[191] Han Y., Tetik H., Malakooti M.H. (2024). 3D soft architectures for stretchable thermoelectric wearables with electrical self-healing and damage tolerance. Adv. Mater..

[192] Yang L., Chen J., He W., Li G., Xie C., Wang W., Han D., Han C.-G., Niu L. (2025). Improved ionic thermoelectric performance of adhesive and self-healing cationic high-entropy gel thermocell. Angew. Chem. Int. Ed..

[193] Mohammed H., Salleh H., Yusoff M. (2007). The transient response for different types of erodable surface thermocouples using finite element analysis. Therm. Sci..

[194] Grech A., Sant T., Farrugia M. (2009).

[195] Buttsworth D.R. (2002). Transient response of an erodable heat flux gauge using finite element analysis. Proc. Inst. Mech. Eng. D: J. Automob. Eng..

[196] Buttsworth D.R., Stevens R., Stone C.R. (2005). Eroding ribbon thermocouples: impulse response and transient heat flux analysis. Meas. Sci. Technol..

[197] Brunner D., LaBombard B. (2012). Surface thermocouples for measurement of pulsed heat flux in the divertor of the alcator C-mod tokamak. Rev. Sci. Instrum..

[198] Ren J., Donovan D., Watkins J., Wang H.Q., Rudakov D., Murphy C., McLean A., Lasnier C., Unterberg E., Thomas D., Boivin R. (2018). The surface eroding thermocouple for fast heat flux measurement in DIII-D. Rev. Sci. Instrum..

[199] Ren J., Donovan D., Watkins J.G., Wang H.Q., Rudakov D., Murphy C., Unterberg E., Thomas D., Boivin R. (2020). Development of Surface Eroding Thermocouples in Small Angle Slot Divertor in DIII-D. IEEE Trans. Plasma Sci..

[200] Ren J., Donovan D.C., Watkins J.G., Wang H.Q., Lasnier C., Looby T., Canik J., Rudakov D., Stangeby P.C., Thomas D., Boivin R. (2022). Measurements of multiple heat flux components at the divertor target by using surface eroding thermocouples (invited). Rev. Sci. Instrum..

[201] Ren J., Donovan D., Watkins J.G., Wang H.Q., Rudakov D., Looby T., Thomas D., Boivin R. (2022). A Method to Identify the Heat Flux From Photons and Neutrals at the Divertor Target. IEEE Trans. Plasma Sci..

[202] Palma M.D., Spolaore M. (2021). Modelling fast response surface thermocouple for plasma facing components. IEEE Sens. J..

[203] Cui Y., Fu P., Wang H., Yin J. (2025). 2025 5th International Conference on Sensors and Information Technology.

[204] Wu W., Haick H. (2018). Materials and wearable devices for autonomous monitoring of physiological markers. Adv. Mater..

[205] Akbar Z.A., Jeon J.-W., Jang S.-Y. (2020). Intrinsically self-healable, stretchable thermoelectric materials with a large ionic seebeck effect. Energy Environ. Sci..

[206] Malik Y.T., Akbar Z.A., Seo J.Y., Cho S., Jang S.-Y., Jeon J.-W. (2022). Self-healable organic–inorganic hybrid thermoelectric materials with excellent ionic thermoelectric properties. Adv. Energy Mater..

[207] Zeng Q., Zhao Y., Lai X., Jiang C., Wang B., Li H., Zeng X., Chen Z. (2022). Skin-inspired multifunctional MXene/cellulose nanocoating for smart and efficient fire protection. Chem. Eng. J..

[208] Ho D.H., Kim Y.M., Kim U.J., Yu K.S., Kwon J.H., Moon H.C., Cho J.H. (2023). Zwitterionic polymer gel-based fully self-healable ionic thermoelectric generators with pressure-activated electrodes. Adv. Energy Mater..

[209] Jiang Q., Wan Y., Qin Y., Qu X., Zhou M., Huo S., Wang X., Yu Z., He H. (2024). Durable and wearable self-powered temperature sensor based on self-healing thermoelectric fiber by coaxial wet spinning strategy for fire safety of firefighting clothing. Adv. Fiber Mater..

[210] Liu L., Zhang D., Bai P., Mao Y., Li Q., Guo J., Fang Y., Ma R. (2023). Strong tough thermogalvanic hydrogel thermocell with extraordinarily high thermoelectric performance. Adv. Mater..

[211] Zhao W., Zheng Y., Huang A., Jiang M., Wang L., Zhang Q., Jiang W. (2024). Metal-halogen interactions inducing phase separation for self-healing and tough ionogels with tunable thermoelectric performance. Adv. Mater..

[212] Long Q., Jiang G., Zhou J., Zhao D., Yu H. (2024). A cellulose ionogel with rubber-like stretchability for low-grade heat harvesting. Research.

[213] Wang X., Jin Y., Fu H., Zhang L., Lee P.C., An C.J. (2025). Self-healing and stretchable tetrameric PVA-CNF-PVP-IL complex ionogel with high-performance ionic thermoelectric properties. J. Mater. Chem. A.

[214] Huang X.-Y., Zhu H.-Q., Li L.-F., Lv T.-C., Li H.-Y., Gu J.-J., Wang W., Xue B., Lei H., Cao Y. (2025). Tough, transparent, self-healing ionogel with exceptional moisture and impact resistance. Chin. J. Polym. Sci..

[215] Cao Z., Liu H., Jiang L. (2020). Transparent, mechanically robust, and ultrastable ionogels enabled by hydrogen bonding between elastomers and ionic liquids. Mater. Horiz..

[216] Sui C., Zhao W., Guo X., Chen X., Wei S., Zhao W., Yan S. (2024). Robust, transparent, self-healable, recyclable all-starch-based gel with thermoelectric capability for wearable sensor. Int. J. Biol. Macromol..

[217] Zhang M., Liu Y., Li J., Wu C., Wang Z., Liu Y., Wei P., Zhao W., Cai K. (2024). Scalable printing high-performance and self-healable Ag2Se/terpineol nanocomposite film for flexible thermoelectric device. Energy.

[218] Wang D., Wang X., Rao W. (2021). Precise regulation of Ga-based liquid metal oxidation. Acc. Mater. Res..

[219] Sun Y.-W., Sun J.-S., Lv K.-H., Liu J.-P., Shi C.-J., Zhang T.-F., Zheng Y.-F., Yan H., Li Y.-C. (2025). Enhancing acrylamide-based polymer performance in high temperature drilling fluid: role of isopentenol polyoxyethylene ether. Pet. Sci..

[220] Lian Y., Chen X., Zhang T., Liu C., Lin L., Lin F., Li Y., Chen Y., Zhang M., Zhou W. (2023). Temperature measurement performance of thin-film thermocouple cutting tool in turning titanium alloy. Ceram. Int..

[221] Ullah I., Ullah K., Zhao L.-F., Zhou Z.-F. (2025). Machine learning-driven multiobjective optimization of a MEMS in-situ thermoelectric seebeck coefficient measurement structure. Measurement.

[222] Zhang Z., Gong S., Ye J., Zhang C., Chen J., Su Z., Wang H., Liu Z., Hai Z. (2026). Deep learning-based calibration of a multi-point thin-film thermocouple array for temperature field measurement. Sensors.

[223] Wang H., Dinwiddie R.B. (2000). Reliability of laser flash thermal diffusivity measurements of the thermal barrier coatings. J. Therm. Spray Technol..

[224] Wang L., Li D.C., Yang J.S., Shao F., Zhong X.H., Zhao H.Y., Yang K., Tao S.Y., Wang Y. (2016). Modeling of thermal properties and failure of thermal barrier coatings with the use of finite element methods: a review. J. Eur. Ceram. Soc..

[225] Guo K., Lu Y., Liu Z. (2025). High-temperature thin-film thermocouple for aero-engines. PLoS One.

[226] Chen Z., Sun Y., Xue Y., Li D., Zhou S. (2023). 2023 10th International Conference on Dependable Systems and Their Applications (DSA).

[227] Serio B., Nika P., Prenel J.P. (2000). Static and dynamic calibration of thin-film thermocouples by means of a laser modulation technique. Rev. Sci. Instrum..

[228] Zhou W., Zhao Y.G., Li W., Qin Q.D., Hu S.W., Tian B. (2006). Degradation formula and working lifetime prediction for high-temperature coating. Appl. Surf. Sci..

[229] Almeida J.B. (1999). Application of weilbull statistics to the failure of coatings. J. Mater. Process. Technol..

[230] Osuchukwu O.A., Salihi A., Ibrahim A., Audu A.A., Makoyo M., Mohammed S.A., Lawal M.Y., Etinosa P.O., Isaac I.O., Oni P.G. (2024). Weibull analysis of ceramics and related materials: a review. Heliyon.

[231] Ma J., Zhang F., Li Q., Lai S., Zhang H., Wang K., Wriggers P. (2025). Life prediction of thermal barrier coatings on turbine blades based on a multiscale approach. Surf. Coat. Technol..

[232] Chen Z., Zhou K., Lu X., Lam Y.C. (2014). A review on the mechanical methods for evaluating coating adhesion. Acta Mech..

[233] Lima C.R.C., Crespo V., Nin J., Clavé G., Dosta S. (2025). Adhesion of thermal barrier coatings: influence of the bond coat application technique. Surf. Coat. Technol..

[234] Hu T.C., Wang J.F., Xi Y.Y., Sun Y.F. (2022). Research on failure mechanism and thermal stress of thin-film thermocouple at high temperature. Key Eng. Mater..

